# Simplified clinical algorithm for identifying patients eligible for same-day HIV treatment initiation (SLATE): Results from an individually randomized trial in South Africa and Kenya

**DOI:** 10.1371/journal.pmed.1002912

**Published:** 2019-09-16

**Authors:** Sydney Rosen, Mhairi Maskew, Bruce A. Larson, Alana T. Brennan, Isaac Tsikhutsu, Matthew P. Fox, Lungisile Vezi, Margaret Bii, Willem D. F. Venter

**Affiliations:** 1 Department of Global Health, Boston University School of Public Health, Boston, Massachusetts, United States of America; 2 Health Economics and Epidemiology Research Office, Department of Internal Medicine, School of Clinical Medicine, Faculty of Health Sciences, University of the Witwatersrand, Johannesburg, South Africa; 3 Department of Epidemiology, Boston University School of Public Health, Boston, Massachusetts, United States of America; 4 Kenya Medical Research Institute, Kericho, Kenya; 5 Henry M. Jackson Foundation Medical Research International, Inc., Nairobi, Kenya; 6 Ezintsha, Wits Reproductive Health and HIV Institute, Department of Internal Medicine, School of Clinical Medicine, Faculty of Health Sciences, University of the Witwatersrand, Johannesburg, South Africa; University of Southampton, UNITED KINGDOM

## Abstract

**Background:**

The World Health Organization recommends "same-day" initiation of antiretroviral therapy (ART) for HIV patients who are eligible and ready. Identifying efficient, safe, and feasible procedures for determining same-day eligibility and readiness is now a priority. The Simplified Algorithm for Treatment Eligibility (SLATE) study evaluated a clinical algorithm that allows healthcare workers to determine eligibility for same-day treatment and to initiate ART at the patient’s first clinic visit.

**Methods and findings:**

SLATE was an individually randomized trial at three outpatient clinics in urban settlements in Johannesburg, South Africa and three hospital clinics in western Kenya. Adult, nonpregnant, HIV-positive, ambulatory patients presenting for any HIV care, including HIV testing, but not yet on ART were enrolled and randomized to the SLATE algorithm arm or standard care. The SLATE algorithm used four screening tools—a symptom self-report, medical history questionnaire, physical examination, and readiness assessment—to ascertain eligibility for same-day initiation or refer for further care. Follow-up was by record review, and analysis was conducted by country. We report primary outcomes of 1) ART initiation ≤28 days and 2) initiation ≤28 days and retention in care ≤8 months of enrollment. From March 7, 2017 to April 17, 2018, we enrolled 600 patients (median [IQR] age 34 [29–40] and CD4 count 286 [128–490]; 63% female) in South Africa and 477 patients in Kenya (median [IQR] age 35 [29–43] and CD4 count 283 [117–541]; 58% female). In the intervention arm, 78% of patients initiated ≤28 days in South Africa, compared to 68% in the standard arm (risk difference [RD] [95% confidence interval (CI)] 10% [3%–17%]); in Kenya, 94% of intervention-arm patients initiated ≤28 days compared to 89% in the standard arm (6% [0.5%–11%]). By 8 months in South Africa, 161/298 (54%) intervention-arm patients had initiated and were retained, compared to 146/302 (48%) in the standard arm (6% [(2% to 14%]). By 8 months in Kenya, the corresponding retention outcomes were identical in both arms (137/240 [57%] of intervention-arm patients and 136/237 [57%] of standard-arm patients). Limitations of the trial included limited geographic representativeness, exclusion of patients too ill to participate, missing viral load data, greater study fidelity to the algorithm than might be achieved in standard care, and secular changes in standard care over the course of the study.

**Conclusions:**

In South Africa, the SLATE algorithm increased uptake of ART within 28 days by 10% and showed a numerical increase (6%) in retention at 8 months. In Kenya, the algorithm increased uptake of ART within 28 days by 6% but found no difference in retention at 8 months. Eight-month retention was poor in both arms and both countries. These results suggest that a simple structured algorithm for same-day treatment initiation procedures is feasible and can increase and accelerate ART uptake but that early retention on treatment remains problematic.

**Trial registration:**

Clinicaltrials.gov NCT02891135, registered September 1, 2016. First participant enrolled March 6, 2017 in South Africa and July 13, 2017 in Kenya.

## Abstract 2

Sydney Rosen and colleagues investigate the performance of a screening algorithm to assess eligibility for rapid HIV treatment initiation.

## Author summary

**Why was this study done?:**

- Both the World Health Organization and many national governments in sub-Saharan Africa now recommend that patients diagnosed with HIV start antiretroviral treatment (ART) as quickly as they can and, if possible, on the same day as their HIV diagnosis, known as “same-day initiation.”
- Despite the recommendations, initiation still usually requires 2–4 clinic visits before medications are dispensed, and there is little guidance on how to implement same-day initiation, in particular on exactly how to determine if a patient is eligible and ready to start treatment and how to provide the specific services required for ART initiation in a single clinic visit.
- In the SLATE study, we evaluated a simple clinical algorithm to guide nurses and other clinical staff on how to offer same-day initiation to most patients while still providing all the care that HIV patients need.

**What did the researchers do and find?:**

- We individually randomized patients coming to three public-sector clinics in South Africa and three in Kenya for an HIV test or pretreatment care to be offered either same-day ART initiation under the SLATE algorithm or regular (standard-of-care) procedures for ART initiation.
- The intervention allowed half of the patients in South Africa and 70% of them in Kenya to initiate ART on the same day (i.e., in a single visit); most who could not had symptoms of tuberculosis (TB) and required a TB test before starting ART.
- The proportions starting treatment within 7 and 28 days of study enrollment increased by 27% and 10% in South Africa and by 13% and 6% in Kenya.
- There was little or no difference in retention in care or viral suppression rates 8 months after study enrollment, with very poor retention observed in both countries.
- Nearly every patient in the study (98%) said that they would like to start treatment on the same day if they could.

**What do these findings mean?:**

- The SLATE study demonstrates that at least half of all HIV-positive patients who come to clinics and are not yet on HIV treatment are eligible and ready for same-day initiation; initiation can safely be done without waiting for laboratory test results, and the vast majority of patients would like this option.
- Common reasons for delaying ART initiation, such as the presence of TB symptoms or providers’ concerns about treatment adherence, should be investigated further because the benefits of offering medications on the day of diagnosis may outweigh some of these risks.
- Loss of patients from care after starting ART remains a major challenge regardless of the manner or speed of initiation.

## Introduction

In July 2017, the World Health Organization (WHO) revised its guidelines for antiretroviral therapy (ART) for HIV to recommend “same-day” treatment initiation (on day of diagnosis) whenever possible and “rapid” initiation (within 7 days of diagnosis) for all HIV-positive patients [1]. The guidelines cited evidence from clinical trials suggesting that offering treatment to patients at their first clinical encounter has the potential to increase relative uptake of ART within 90 days by 30% and from observational studies that showed an overall relative increase of 53%. These studies varied widely from one another in the clinical approaches used, intervention design (same-day initiation or rapid initiation), and the populations studied [1].

Both WHO’s new guidelines [1] and national guidelines in South Africa [2] and Kenya [3] recommend same-day initiation if the patient is “ready” (WHO), “clinically ready and willing to commit” (South Africa), or “as soon as patient is ready” (Kenya). Little guidance is provided, however, on exactly how to determine if a patient is ready to start treatment or how to provide the specific services required for ART initiation in a single clinic visit.

All of the trials cited by WHO relied on point-of-care (POC) instruments, which are not feasible in most routine care settings. A cost-effectiveness analysis of one of them, the RapIT trial in South Africa [4], found that the POC instruments used increased the cost per patient initiated substantially [5], and requirements for power, internet access, maintenance, and quality assurance make scale-up of POC instruments infeasible in most low-resource settings. In late 2015, a technical consultation to develop a post-RapIT research agenda on how to accelerate ART initiation proposed a clinical algorithm intended to allow nurses and other clinicians to determine eligibility for same-day initiation and start ART without relying on POC tests or waiting for laboratory results, using a comprehensive, standardized algorithm [6]. The Simplified Algorithm for Treatment Eligibility (SLATE) trial evaluated a refined version of that algorithm, powered separately in South Africa and Kenya. The study’s goal was to determine whether an algorithm for same-day ART initiation that can be implemented in routine care settings without reliance on laboratory results can safely and effectively increase and accelerate uptake of ART in the general adult population. We report primary and secondary outcomes, including ART initiation within 7 and 28 days and retention in care at 8 months after study enrollment.

## Methods

### Study design

**Fig 1:**
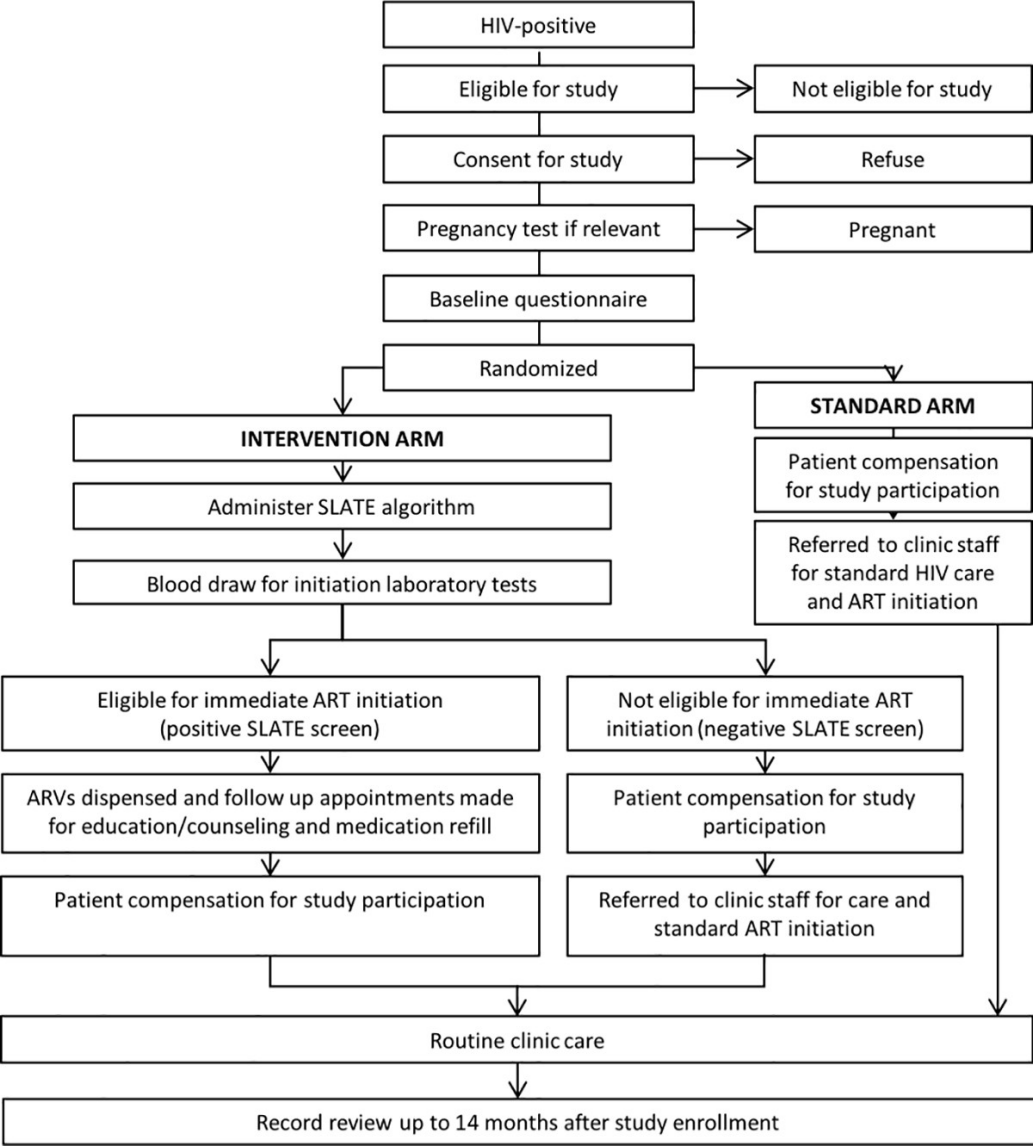
Study procedures for the SLATE randomized controlled trial. ART, antiretroviral therapy; SLATE, Simplified Algorithm for Treatment Eligibility.

SLATE was an unblinded, individually randomized trial of an intervention that allows clinicians to determine eligibility for same-day initiation and dispense antiretroviral medications (ARVs) at any clinic visit, including the first visit for an HIV test, using a clinical algorithm that does not require laboratory test results prior to initiation and can be implemented by typical clinic staff. It received ethics approval from the institutional review boards of Boston University (BUMC H-35634), the University of the Witwatersrand (HREC 160910), and the Kenya Medical Research Institute (SERU 3408) and is registered with ClinicalTrials.gov, number NCT02891135. Study procedures have been described in detail previously [7] and are illustrated in Fig 1. The research protocol is included as S1 Text and the CONSORT checklist as S1 Checklist.

During the period of study enrollment (March, 2017–April, 2018), all HIV-positive individuals were eligible for ART under South Africa’s and Kenya’s universal treatment guidelines, regardless of CD4 count. Under standard care, guidelines called for a preinitiation CD4 count, creatinine clearance test, and hemoglobin in both countries and alanine aminotransferase in South Africa. Guidelines also recommended that patients with symptoms of tuberculosis (TB; any cough, fever, night sweats, or weight loss) be asked for a sputum sample, to be tested at a centralized (South Africa) or on-site (Kenya) laboratory using Xpert MTB/RIF. Blood samples from patients with CD4 counts ≤100 cells/mm^3^ were reflexively tested for cryptococcal antigen (CrAg). Treatment-naïve patients were initiated on the standard first-line ARV regimen of tenofovir, emtricitabine (South Africa)/lamivudine (Kenya), and efavirenz, dispensed in a combined once-daily tablet. After initiation, patients in South Africa were asked to return to the clinic for monitoring at 1, 2, 3, 6, and 12 months, and 6-monthly thereafter [8]; patients in Kenya were asked to return at 2 weeks and 4 weeks and then monthly until virally suppressed, with visits every 3 months thereafter. Medication refill visit schedules depended on inventories available and provider judgment, with a 1–3 months’ supply typically dispensed at each visit.

### Study sites, infrastructure, and staffing

SLATE was conducted at a convenience sample of three public-sector primary care clinics serving densely settled, urban formal and informal populations around Johannesburg, South Africa and three public-sector HIV outpatient clinics located within county hospitals in western Kenya. All study sites received some level of support from nongovernmental partners of the U.S. President’s Emergency Plan For AIDS Relief (PEPFAR), as was typical of most large facilities in both countries. Details of infrastructure and staffing varied by country. All study staff completed ethics and study-specific training.

In South Africa, an interview room and an examination room located either in the clinic building or in a mobile trailer on the clinic grounds were designated for study procedures and storage of study equipment and supplies. Clinical procedures and administration of the algorithm among those randomized to the intervention arm were performed by study nurses with the same clinical qualifications as existing primary healthcare nurses responsible for ART initiation. Nonclinical procedures (recruitment, consent, questionnaire, patient flow management, data capturing onto mobile tablets) were implemented by study assistants, some of whose qualifications were comparable to those of experienced lay counselors at the sites.

In Kenya, an interview room and an examination room were located in clinic buildings at all three sites. Clinical procedures and administration of the algorithm were performed by the same clinical officers who were already responsible for ART initiation, with one per site employed part-time by the study. Nonclinical procedures (recruitment, consent, questionnaire, patient flow management, data capturing onto mobile tablets) were implemented by study assistants who were trained clinic nurses, again working on a part-time basis for the study.

### Study population

The study enrolled adult (≥18 years old), nonpregnant, HIV-positive patients not yet on ART who presented at one of the study clinics to have an HIV test, enroll in care or prepare to start or restart ART if already diagnosed, or receive other unrelated medical care that led to referral for an HIV test. Pregnant women were excluded because standard-of-care prevention of mother-to-child transmission (PMTCT) is typically provided by the antenatal clinic rather than the general ART clinic. During screening, patients who were unwilling to hear about the study, were currently on ART or had been dispensed ARVs in the preceding 90 days, indicated that they planned to seek HIV care during the next 12 months at a different clinic, or were judged by clinic or study staff to be physically or emotionally unable to provide consent or participate in all study procedures were excluded. Female patients found postenrollment to be pregnant were withdrawn prior to randomization and referred for antenatal ART initiation.

### Enrollment

After obtaining written informed consent, each study participant was assigned a unique study ID that was electronically scanned and used to link all electronic forms captured on mobile tablets in REDCap Mobile [9]. The study assistant then administered a questionnaire to all study participants asking questions about the patients’ demographic characteristics, HIV history and treatment preferences, employment and primary activities, and visit costs.

Each participant was offered compensation for participation equivalent in value to US$5–$15, in the form of a shopping voucher that could be used at nearby grocery/general goods stores in South Africa and cash in Kenya, as illustrated in Fig 1.

### Randomization

Participants were individually randomized 1:1 to the intervention arm or to standard of care using block randomization in blocks of six. Allocations were generated by MM using a computerized random-number generator under oversight of the principal investigator and numbered sequentially. They were then placed in opaque envelopes and sealed by ATB. The envelopes were kept in sequential, numbered order at the study sites, with an equal number distributed to each site to balance enrollment by site. On completion of the questionnaire, the study assistant opened the next sequentially numbered randomization envelope to reveal the patient’s allocation. No blinding of patients or providers was possible because each arm entailed different procedures and staff.

### Standard-arm procedures

Study staff interaction with participants in the standard arm was limited to screening for study eligibility, obtaining written informed consent, administering a questionnaire, and referral back to standard care. Standard-arm patients were accompanied to the appropriate location in the clinic (e.g., counselor station, registration desk, or TB room) to continue with a standard care visit. After referral, patients in the standard arm were followed passively through medical record review and had no further interaction with study staff.

Standard-of-care procedures for ART initiation generally followed national guidelines in each country as outlined below, though exact procedures varied somewhat by site. During the period of study enrollment in South Africa (2017), guidelines recommended ART initiation “as soon as the patient is ready and within two weeks of CD4 count being done” for most patients and within 1 week for those presenting very ill [8,10]. A national evaluation conducted in South Africa in 2017 reported that an average of three visits to a clinic were still required before ARVs were dispensed: one visit for an HIV test, TB symptom screen, and sputum sample if symptomatic, and an initial adherence session; a second visit for remaining adherence sessions and return of laboratory results; and a third visit for a clinical examination and dispensing of ARVs. These visits were typically completed over a 2–4 week period [11,12]. Patients with positive TB test results or other conditions entailing additional care required more visits over a longer period of time. All laboratory tests were processed at centralized public-sector laboratories, with results available at the patient’s next visit. Patients who had already completed some preinitiation steps, such as an HIV test and blood draw for a CD4 count, at the time of study enrollment required few or no additional visits under standard care, such that some patients enrolled in the study could be dispensed ARVs by clinic staff on the day of study enrollment because they had already completed all preinitiation steps.

In Kenya, standard-of-care guidelines at the time of SLATE I enrollment recommended that all patients initiate within 2 weeks of HIV care enrollment but allowed same-day initiation for those thought to have “strong motivation” [13]. An informal prestudy review of clinic records suggested that the use of same-day initiation varied by site but was quite common overall. During study enrollment, the standard-of-care ART initiation process required an HIV test (if not already done) and confirmatory HIV test, a complete medical and psychosocial history, a thorough physical exam, HIV-specific and nonspecific laboratory investigations (but not as a prerequisite to ART initiation), screening for TB, and a variety of other assessments and counseling activities addressing reproductive health, noncommunicable diseases, mental health, nutrition, alcohol and substance abuse, and education on HIV and its treatment.

### Intervention-arm procedures

**Fig 2:**
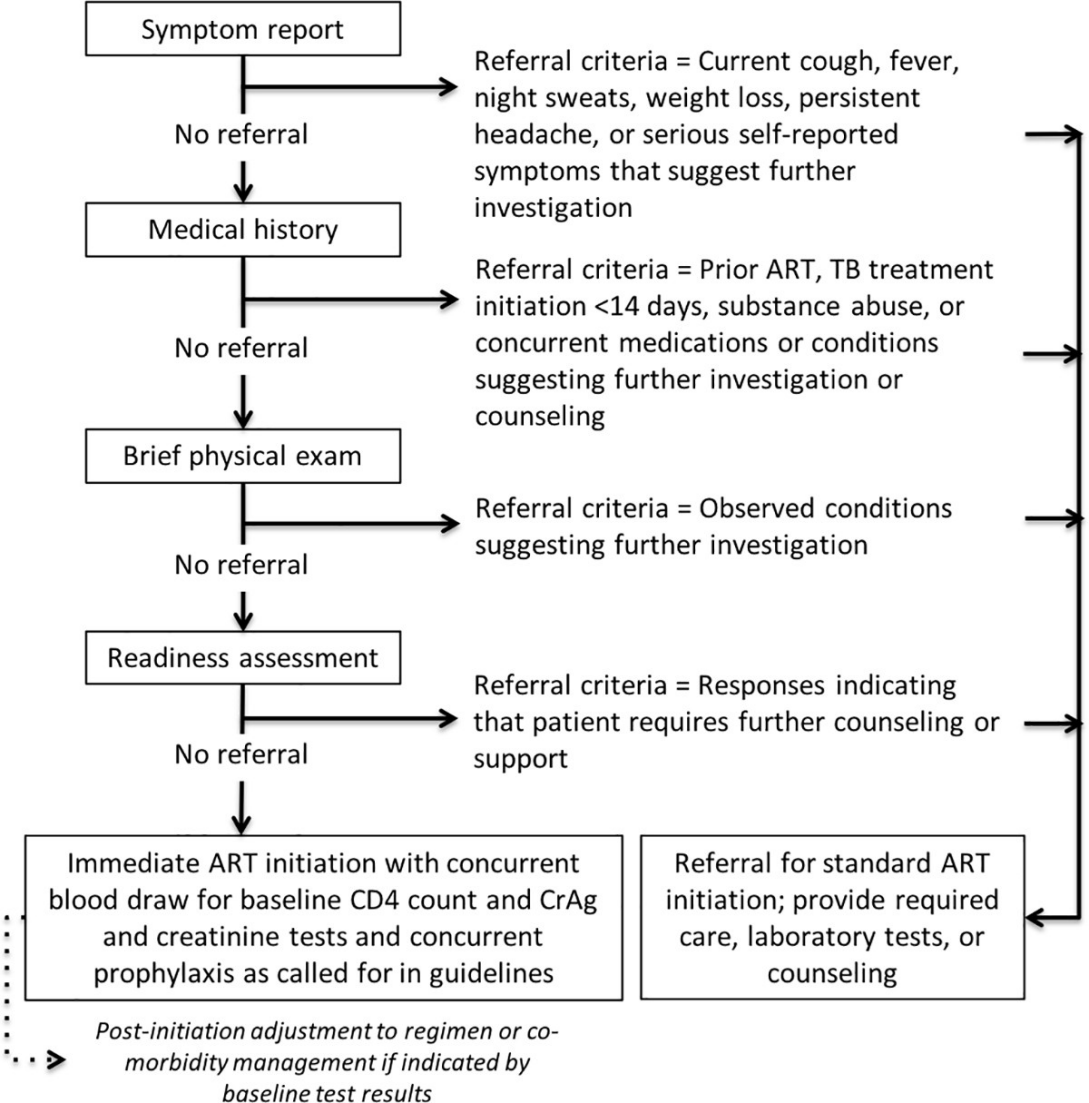
SLATE algorithm to support same-day HIV treatment initiation. ART, antiretroviral therapy; CrAg, cryptococcal antigen; SLATE, Simplified Algorithm for Treatment Eligibility; TB, tuberculosis.

For patients randomized to the intervention arm, the SLATE algorithm (Fig 2 and S1 Table) was administered by a study nurse in South Africa and a clinical officer in Kenya. The algorithm comprised four “screens”: symptom report, medical history, physical examination, and readiness assessment. Each screen was designed to identify clinical, historical, or personal reasons for which a patient should be referred for additional care, investigations, or services needed before ART could be initiated without compromising patient welfare.

For patients who “screened in” under the algorithm—i.e., did not report or demonstrate any reason to delay ART initiation—28 days/14 days of medication was dispensed by the study clinician in South Africa/Kenya, and the patient was accompanied to the clinic booking office to schedule their next clinic appointment. For those who “screened out” on any one of the four SLATE algorithm screens, indicating at least one condition or concern that suggested additional services were needed, the study clinician referred the patient back to the site for further clinical investigation or care following the site’s routine procedures as warranted. All patients who screened out of same-day initiation in the intervention arm were offered a referral letter to give to the site clinic detailing the condition or concern reported by the study nurse. After the study enrollment visit, all patients received follow-up care from clinic staff under routine procedures. The previously published protocol for the study provides further details about procedures [7].

### Data collection

Data were collected from four sources. First, at the study enrollment visit, a case report form (CRF) was completed, with eligibility and questionnaire data for all participants and SLATE algorithm data for intervention-arm participants. CRF data were entered by study staff onto tablet computers programmed for data collection using REDCap Mobile [9]. Second, baseline blood tests (e.g., CD4 counts) and TB test results were extracted directly from laboratory electronic records or paper-based registers kept at each site. Third, follow-up data for the period from enrollment to study endpoints were collected from routinely generated clinical record data from patient records in electronic and paper format. And fourth, viral load test results were obtained from national laboratory databases maintained by the National Health Laboratory Service in South Africa and the National AIDS & STI Control Programme (NASCOP) in Kenya. Viral load outcomes were thus not limited to tests originating at the study sites, though all other data were. Further details about data sources and quality are provided in S2 Text.

Patients had no personal interaction with the study team after the enrollment visit because all follow-up was based on record review only. Study patients received no support for retention or adherence from the study. Clinics varied in their efforts to trace patients lost to follow-up, but we observed little active tracing during the study period.

### Study outcomes

The primary outcomes of the study were 1) ART initiation ≤28 days of study enrollment and 2) ART initiation ≤28 days and retention in care 8 months after study enrollment (both conditions had to be met to achieve this outcome). Previous studies have found that 28 days is a sufficient time interval for a majority of patients found eligible for ART to complete the steps required to start treatment under routine care [10,13]. We note that 28 days was a relatively generous interval to allow for achievement of this outcome since standard care guidelines at the time of study enrollment called for initiation within 14 days [14,15]. In light of the recent WHO recommendation for “rapid” initiation of all patients, defined as initiation within 7 days, we also report this additional outcome alongside the original primary outcomes.

The 8-month interval allowed patients up to 1 month (28 days) for ART initiation, 6 months to reach the routine 6-month clinic visit called for by guidelines, and 1 additional month for the 6-month visit to be completed. We defined a patient as “retained” if the patient initiated within 28 days of enrollment and a clinic visit was made or a viral load test observed between 5 and 8 months after enrollment, allowing a broad window for irregular visit schedules. We reviewed patients’ records for up to 3 months after the 8-month outcome window to ensure that we captured information generated within 8 months but only recorded in the EMR or paper files up to 3 months later. Patients who were not retained were reported as known to have died, known to have transferred care to another facility, or, most often, lost to follow-up from the study site (i.e., did not have visit or test 5–8 months after enrollment).

Secondary outcomes reported here include time to initiation in days, proportion of patients initiating within 7 and 14 days, proportion of patients who screened in and out of the SLATE algorithm, reasons for screening out, and self-reported patient preferences on the timing of ART initiation, using baseline questionnaire data. We also report the secondary outcome of viral suppression (<400 copies/mL) between months 5 and 8 after study enrollment, conditional on achieving the 8-month primary outcome, to capture the routine 6-month viral load test called for in national guidelines. We found, however, that many patients who achieved the study’s 8-month outcome had not had a viral load test by 8 months. Viral load results were thus missing for a large number of patients.

### Sample size

The study was designed to detect an absolute increase of 15% in patients achieving our second primary outcome from an estimated baseline of 65%, as observed in the RapIT trial [4], to an intervention outcome of 80%. With an α of 0.05, power of 90%, 1:1 randomization, and an uncorrected Fisher’s exact test, we estimated that we would need to enroll at least 197 patients per arm, which we increased to a maximum of 240 per arm to ensure sufficient power after accounting for anticipated postconsent withdrawal and ineligibility. In South Africa, the sample size was further increased to a maximum of 330 per arm (660 in total). We suspected fairly early in the study that results would vary quite a lot by site, particularly in South Africa. Increasing the sample size facilitated analysis of effect modification by site.

### Data analysis

Characteristics at study enrollment of all randomized participants were summarized using simple proportions and medians with interquartile ranges (IQRs), stratified by treatment arm. We compared the proportions of patients achieving each dichotomized study outcome and present crude risk differences (RDs) and crude relative risks (RRs) with 95% confidence intervals (CIs) stratified by group. RDs were estimated using a linear probability model with robust standard errors. RRs were estimated using a log-linear generalized linear model, also with robust standard errors. All analyses were by modified intention to treat: patients randomized to the intervention arm who screened out of immediate ART initiation under the SLATE algorithm remained in the intervention arm for data analysis, with the exception of participants who were excluded because there was not enough time for them to complete study procedures by the end of the day. We looked for absolute effect modification by important predictors of each outcome: age, sex, site, CD4 count, and reason for clinic visit. We used a simple stratification of the primary analysis by the potential modifier and report crude RDs and risk ratios and their corresponding 95% CIs.

## Results

**Fig 3:**
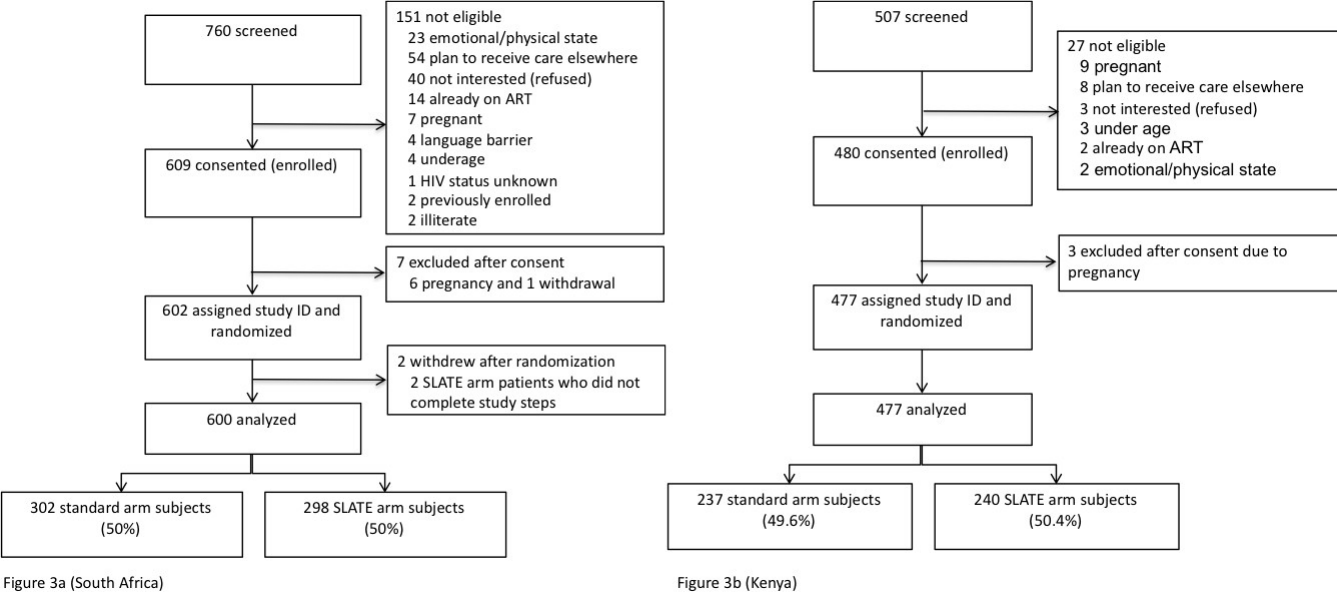
CONSORT flow diagram for the SLATE randomized trial of same-day treatment initiation. (a) South Africa; (b) Kenya. ART, antiretroviral therapy; SLATE, Simplified Algorithm for Treatment Eligibility.

During the South Africa study enrollment period from March 6, 2017 to July 28, 2017, 760 patients were screened for study eligibility (Fig 3A). Of these, 609 were eligible and provided written informed consent. Of the 151 screened who did not meet study eligibility criteria, 54 intended to seek further care elsewhere, and 40 refused participation. Other reasons for ineligibility are shown in Fig 3A. Six female patients had positive pregnancy tests after consent and were excluded, and one patient withdrew after consenting, making the total randomized 602. After randomization, two patients were unable to complete study procedures on the day of enrollment because of lack of time and were removed from further analyses, leaving 600 patients in the final South Africa analytic cohort. Of these, 302 were randomized to the standard arm and 298 to the intervention arm. Follow-up continued through June 30, 2018, when all participants had reached the 8-month follow-up interval at which the primary outcome was assessed.

During the Kenya study enrollment period from July 13, 2017 to April 17, 2018, 507 patients were screened for study eligibility (Fig 3B). Of these, 480 were eligible and provided written informed consent. Of the 27 screened who did not meet study eligibility criteria, nine were pregnant, and eight intended to seek further care elsewhere. Other reasons for ineligibility are shown in Fig 3B. Three female patients had positive pregnancy tests after consent and were excluded, making the total randomized and remaining in the analytic cohort 477. Of these, 237 were randomized to the standard arm and 240 to the intervention arm. Follow-up continued through December 23, 2018, when all participants had reached the 8-month interval for the primary outcome.

### Study population characteristics

Baseline demographic, clinical, and economic characteristics of participants stratified by study arm at time of enrollment are reported in Table 1. A majority of participants (63% in South Africa and 58% in Kenya) were female; the median ages were 34 and 36 years, respectively, and median baseline CD4 counts were 277 cells/mm^3^ (IQR 141–484) and 283 cells/mm^3^ (IQR 117–541). As Table 1 indicates, there were small differences in baseline CD4 count strata between arms in both countries, but medians and IQRs were similar. In South Africa, more intervention-arm patients than standard-arm patients presented with CD4 counts ≤100 (20% versus 15%), but the proportion with CD4 counts <200 was similar between the arms (36% versus 35%). In Kenya, 39% of standard-arm patients were missing CD4 count results, mainly because of the site laboratories’ equipment failures or lack of reagents. There were small differences between the study arms in each country for some other variables, but none that appear meaningful for interpretation of trial results.

**Table 1:** Patient characteristics.

		South Africa		Kenya	
		Standard arm (N = 302)	Intervention arm (N = 298)	Standard arm (N = 237)	Intervention arm (N = 240)
Study site	South Africa Site 1: City of Johannesburg	125 (41%)	124 (42%)		
	South Africa Site 2: Ekurhuleni District	90 (30%)	90 (30%)		
	South Africa Site 3: City of Johannesburg	87 (29%)	84 (28%)		
	Kenya Site 1: Kericho			90 (38%)	90 (38%)
	Kenya Site 2: Kapsabet			78 (33%)	81 (34%)
	Kenya Site 3: Kombewa			69 (29%)	69 (29%)
Sex	Female	190 (63%)	189 (63%)	134 (57%)	142 (59%)
Age (years)	Median (IQR)	34 (28–40)	34 (29–41)	35 (29–44)	36 (29–44)
CD4 count (cells/mm^3^)	Sample rejected	4/302 (1%)	5/298 (2%)	0/237 (0%)	0/240 (0%)
	Missing	29/302 (10%)	5/298 (2%)	92/237 (39%)	19/240 (8%)
	Patients with valid CD4 count result (n)	269/302 (89%)	288/298 (97%)	145/237 (61%)	221/240 (92%)
	Median (IQR)	296 (150–504)	275 (132–459)	297 (94–577)	272 (124–522)
	<100	40 (15%)	60 (20%)	38 (16%)	46 (19%)
	100–200	54 (20%)	45 (16%)	19 (8%)	36 (15%)
	201–350	60 (22%)	69 (24%)	21 (9%)	52 (22%)
	351–500	47 (18%)	54 (19%)	23 (10%)	27 (11%)
	>500	68 (25%)	60 (21%)	44 (19%)	60 (25%)
Location patient currently resides in	Informal urban	263 (87%)	266 (89%)	73 (31%)	66 (28%)
	Urban	27 (9%)	18 (6%)	30 (13%)	35 (15%)
	Rural	12 (4%)	14 (5%)	134 (57%)	139 (58%)
Current house is primary residence	Yes	152 (50%)	141 (47%)	145 (61%)	154 (64%)
Marital status	Single	189 (63%)	178 (60%)	44 (19%)	49 (20%)
	Married or long-term partner	94 (31%)	93 (31%)	126 (53%)	118 (49%)
	Divorced or widowed	19 (6%)	27 (9%)	67 (28%)	73 (30%)
Number of other persons in house	Median (IQR)	2 (1–3)	2 (1–3)	3 (1–5)	3 (2–5)
Usual activity patient does when well	Formal employment	77 (25%)	82 (28%)	21 (9%)	18 (8%)
	Informal sector work	78 (26%)	80 (27%)	169 (71%)	161 (67%)
	Unemployed, looking for work	109 (36%)	107 (36%)	11 (5%)	12 (5%)
	Other	38 (12%)	29 (10%)	36 (15%)	48 (20%)
Previously visited this clinic for any reason	No	204 (68%)	210 (70%)	192 (81%)	195 (81%)
	Yes	97 (32%)	88 (30%)	45 (19%)	45 (19%)
	Missing	1 (0%)	0 (0%)	0 (0%)	0 (0%)
Reason for today's visit	HIV test	162 (54%)	148 (50%)	109 (46%)	114 (48%)
	HIV care (diagnosed previously)	133 (44%)	133 (45%)	97 (41%)	98 (41%)
	Other reason	7 (2%)	17 (5%)	73 (31%)	74 (31%)
Year first tested positive for HIV	Never tested before today	166 (55%)	170 (57%)	144 (61%)	148 (62%)
	2016 or earlier	75 (25%)	79 (27%)	26 (11%)	36 (15%)
	During 2017	61 (20%)	49 (16%)	54 (23%)	36 (15%)
	During 2018	n.a.	n.a.	13 (5%)	20 (8%)
Previously told eligible for ART	No	202 (67%)	199 (67%)	152 (64%)	159 (66%)
	Yes	99 (33%)	99 (33%)	85 (36%)	81 (34%)
	Missing	1	0	0	0
Transport mode to clinic today	Private car	16 (5%)	19 (6%)	15 (6%)	11 (5%)
	Taxi or matatu (public/private minibus)	122 (40%)	122 (41%)	109 (46%)	118 (49%)
	Motorcycle taxi	0 (0%)	0 (0%)	126 (53%)	137 (57%)
	Walked	164 (54%)	156 (53%)	59 (25%)	46 (19%)
	Missing	0	1	0	0
Paid to travel to clinic today	Yes	133 (44%)	134 (45%)	185 (78%)	207 (86%)
Travel cost one way ($US 2018*) if any	Median (IQR)	$0.70 (0.50–0.70)	$0.50 (0.50–0.70)	$0.70 (0.50–1.10)	$0.70 (0.50–1.00)
Travel time one way (min)	Median (IQR)	15 (10–30)	15 (10–30)	30 (20–45)	30 (20–50)
Expected to start ART today	No	113 (38%)	107 (36%)	125 (53%)	113 (47%)
	Yes	188 (62%)	190 (64%)	112 (47%)	127 (53%)
	Missing	1 (0%)	1 (0%)	0 (0%)	0 (0%)
Have enough information to start ART today	Yes	193 (64%)	174 (58%)	179 (76%)	186 (78%)
When would patient want to start ART?	Today	282 (93%)	286 (96%)	231 (97%)	235 (98%)
	Within a week	13 (4%)	7 (2%)	4 (2%)	3 (1%)
	Within a month	4 (1%)	2 (1%)	1 (0%)	1 (0%)
	Not ready	3 (1%)	3 (1%)	1 (0%)	1 (0%)

*Local currencies converted to U.S. dollars using the 2018 average exchange rate reported at http://data.imf.org/?sk=4C514D48-B6BA-49ED-8AB9-52B0C1A0179B (1 U.S. dollar = 101.3 Kenya shillings and 13.24 South African rand).

**Abbreviations:** ART, antiretroviral therapy; IQR, interquartile range; n.a., not applicable.

In South Africa, 69% of the study sample said that they had never been to that clinic for any reason before the day of study enrollment, and 52% reported that one of the reasons for coming to the clinic on the day of study enrollment was to test for HIV. In Kenya, 81% of subjects said that they had never been to that clinic for any reason before the day of study enrollment, and 47% reported that one of the reasons for coming to the clinic on the day of study enrollment was to test for HIV. In both countries, well over 90% of patients indicated that they would want to start ART on the same day if they could, regardless of the reason for their clinic visit.

### ART initiation within 28 days and timing of initiation

As reported in Table 2, initiation of ART within 0, 7, 14, and 28 days of study enrollment was higher in the intervention arm in both countries. In South Africa, 78% (232/298) of intervention-arm patients were documented to have initiated ART within 28 days, compared to 68% (204/302) of patients in the standard arm, for an absolute RD of 10% (3%–17%). In Kenya, 94% (226/240) of intervention-arm patients were documented to have initiated ART within 28 days, compared to 89% (210/237) of patients in the standard arm, for an RD of 6% (1%–11%).

**Table 2:** Initiation, retention, and viral suppression.

Outcome	South Africa				Kenya			
	Standard (n = 302)	Intervention (n = 298)	Crude RD (95% CI)*	Crude RR (95% CI)*	Standard (n = 237)	Intervention (n = 240)	Crude RD (95% CI)*	Crude RR (95% CI)*
***Initiation ≤28 days***								
Initiated ≤28 days	204 (68%)	232 (78%)	10% (3%–17%)	1.15 (1.04–1.27)	210 (89%)	226 (94%)	6% (1%–11%)	1.06 (1.01–1.12)
Record found, did not initiate ≤28 days	78 (25%)	52 (17%)			27 (11%)	14 (6%)		
No record found, did not initiate ≤28 days	20 (7%)	14 (5%)			0 (0%)	0 (0%)		
***Time to initiation***								
Initiated in 0 days (same day)	33 (11%)	161 (54%)	43% (36%–50%)	4.94 (3.52–6.94)	127 (54%)	167 (70%)	16% (7%–25%)	1.30 (1.12–1.50)
Initiated within 7 days (“rapid”)^†^	114 (38%)	193 (65%)	27% (19%–35%)	1.72 (1.45–2.03)	173 (73%)	207 (86%)	13% (6%–20%)	1.18 (1.08–1.30)
Initiated within 14 days^‡^	170 (56%)	207 (69%)	13% (6%–20%)	1.23 (1.09–1.40)	201 (85%)	217 (90%)	6% (0%–12%)	1.07 (1.00–1.14)
Initiated within 90 days^†^	238 (79%)	256 (86%)	7% (0%–11%)	1.09 (1.01–1.17)	222 (94%)	231 (96%)	3% (–1 to 7%)	1.03 (0.99–1.07)
No record of initiation ≤90 days	64 (21%)	42 (14%)	–7% (–13% to 1%)	0.67 (0.47–0.95)	15 (6%)	9 (4%)	–3% (–7% to 1%)	0.60 (0.26–1.33)
***Retained in care by 8 months***								
Initiated ≤28 days and retained 8 months after enrollment	146 (48%)	161 (54%)	6% (–2% to 14%)	1.12 (0.96–1.31)	136 (57%)	137 (57%)	–1% (–8% to 10%)	1.02 (0.87–1.19)
*Of those who did not achieve this outcome*:								
Record found, did not initiate ART ≤28 days	78 (26%)	52 (17%)	–8% (–15% to –2%)	0.68 (0.49–0.92)	27 (11%)	14 (6%)	–5% (–10% to 0%)	0.5 (0.28–0.98)
Record found, initiated ART but lost to follow-up by 8 months	47 (16%)	53 (18%)	3% (–3% to 10%)	1.14 (0.80–1.64)	63 (27%)	65 (27%)	0% (–7% to 8%)	1.02 (0.76–1.37)
Record found, initiated ART but transferred to new provider	4 (1%)	11 (4%)	3% (0%–5%)	2.79 (0.90–8.65)	7 (3%)	17 (7%)	4% (0%–8%)	2.40 (1.01–5.68)
Record found, initiated ART but deceased	1 (0%)	2 (1%)	0% (0%–1%)	2.03 (0.18–22.23)	4 (2%)	7 (3%)	1% (–1% to 4%)	1.73 (0.51–5.83)
Record not found (either before or after initiation)	26 (9%)	19 (6%)	2% (–6% to 2%)	0.74 (0.42–1.31)	0 (0%)	0 (0%)	0% (0%–0%)	1.0 (1.0–1.0)
***Viral suppression at 8 months after enrollment among those who achieved initiation ≤28 days and retention at 8 months***								
N	146	161			136	137		
Record found, virally suppressed	90 (62%)	93 (58%)	–4% (–15% to 7%)	0.94 (0.78–1.13)	94 (69%)	88 (64%)	–5% (–2% to 6%)	0.93 (0.79–1.10)
*Of those who did not achieve this outcome*:								
Record found, virally unsuppressed	7 (5%)	6 (4%)	–1% (–6% to 3%)	0.78 (0.27–2.26)	12 (9%)	5 (4%)	–5% (–11% to 0%)	0.41 (0.15–1.14)
Remained in care but record not found	49 (34%)	62 (39%)	5% (–6% to 16%)	1.15 (0.85–1.55)	30 (22%)	44 (32%)	10% (0%–21%)	1.46 (0.98–2.17)

*Reference group: standard arm.

^†^Post hoc outcome.

^‡^Secondary outcome.

**Abbreviations:** ART, antiretroviral therapy; CI, confidence interval; RD, risk difference; RR, relative risk.

Within 7 days of study enrollment—the WHO definition of “rapid” initiation—65% (193/298) of intervention-arm patients and 38% (114/302) of standard-arm patients in South Africa had started treatment, for an RD of 27% (19%–35%). An additional 24 (8%) patients in the intervention arm and 34 (11%) patients in the standard arm initiated between 28 and 90 days after enrollment. By 3 months after study enrollment, there was still no record of ART initiation for 14% of intervention-arm patients and 21% of standard-arm patients (–7% [–13% to 1%]).

In Kenya, 86% (207/240) of intervention-arm patients and 73% (173/237) of standard-arm patients had started treatment by 7 days, for an RD of 13% (6 to 20%). An additional 5 (2%) patients in the intervention arm and 12 (5%) patients in the standard arm initiated between 28 and 90 days after enrollment. By 3 months after study enrollment, there was still no record of ART initiation for 4% of intervention-arm patients and 6% of standard-arm patients (–3% [–7% to 1%]).

Finally, just over half of intervention-arm patients (161/298) in South Africa initiated on the same day as study enrollment, as did 11% of standard-arm patients (33/302). Of the 149 intervention-arm patients screened out of same-day initiation, cumulative numbers initiating within 0, 7, 14, and 28 days were 12 (8%), 44 (30%), 58 (39%), and 83 (56%), respectively; 52 patients screened out of same-day initiation did not initiate within 28 days, and no records were found for the remaining 14 patients.

In Kenya, 70% (167/240) of intervention-arm patients initiated on the same day as study enrollment, as did 54% of standard-arm patients (127/237). A total of 109 patients randomized to the intervention were deemed ineligible to received same-day treatment through the SLATE I study; of these patients, cumulative numbers initiating within 0, 7, 14, and 28 days were 41 (38%), 78 (72%), 86 (79%), and 95 (87%), respectively; 6 patients screened out of same-day initiation did not initiate within 28 days, and no records were found for the remaining 8 patients.

### Retention at 8 months

The second protocol-defined primary outcome was initiation by 28 days and retained in care 8 months after study enrollment, as indicated by a clinic visit or observed laboratory test between 5 and 8 months after enrollment. In the intervention arm in South Africa, 161 of 298 (54%) patients achieved the second primary outcome, compared to 146/302 (48%) in the standard arm, showing a numerical 6% [–2% to 14%] increase in absolute risk. Results were nearly identical between arms in Kenya: 57% of patients in each study arm achieved the second primary outcome.

### Viral suppression within 8 months of enrollment

As defined by the study, viral suppression as an outcome pertains only to the subset of patients who achieved the second primary outcome (initiated within 28 days and retained at 8 months’ postenrollment). In South Africa and Kenya, 60% and 67% of these patients had viral load test results reported in their records by 8 months after enrollment and were virally suppressed, respectively (Table 2). We found no difference in known viral suppression by 8 months among these patients.

### Algorithm results

In South Africa, among the 298 patients in the intervention arm, exactly half (n = 149) were eligible for same-day initiation according to the SLATE algorithm. Among the remaining 149 patients who screened out of same-day initiation—many for multiple reasons—109 (73%) had one or more symptoms of TB, 17 (11%) reported persistent headache, 14 (9%) had previously defaulted ART, 6 (4%) said they were not ready, 6 (4%) reported substance abuse issues, and 6 (4%) presented with a concerning clinical condition unrelated to TB or other serious opportunistic infection. Among the 52 intervention-arm patients who screened out of same-day initiation and did not initiate within 28 days, 33 (63%) had TB symptoms, 3 (6%) had persistent headache, 7 (13%) were previous defaulters, 2 (4%) reported substance abuse, and 7 (13%) were not ready to start. A further 12 patients (8%) who screened out of same-day ART in the intervention arm were initiated on the day of enrollment by clinic staff following study referral, resulting in a total of 161 intervention-arm patients who initiated ART on the day of study enrollment. The 12 patients initiated by the clinic had screened out of the SLATE algorithm because of TB symptoms (n = 9), headache (n = 2), or substance abuse (n = 1).

In Kenya, among the 240 patients in the intervention arm, 131 (55%) were eligible for same-day initiation according to the SLATE algorithm. Among the remaining 109 patients who screened out of same-day initiation—many for multiple reasons—93 (85%) had one or more symptoms of TB, 31 (28%) reported persistent headache, 18 (17%) had previously defaulted ART, 3 (3%) said they were not ready, 12 (11%) reported substance abuse issues, and 7 (6%) presented with a concerning clinical condition unrelated to TB or another serious opportunistic infection. Among the 14 intervention-arm patients who screened out of same-day initiation and did not initiate within 28 days, 14 (100%) had TB symptoms, 4 (29%) had persistent headache, 1 (7%) was a previous defaulter, 2 (14%) reported substance abuse, and 1 (7%) was not ready to start. Forty-one of 109 (38%) patients who screened out of same-day ART in the intervention arm were initiated on the day of study enrollment by clinic staff following study referral. The 41 patients initiated by the clinic had screened out of the SLATE algorithm because of TB symptoms (n = 30), headache (n = 8), and/or substance abuse (n = 3).

### Absolute effect modification by key variables

Secondary outcomes included an analysis of absolute effect modification by selected variables (S2 Table). We note that the study was not powered to identify effect modifications, and results of this analysis should be interpreted as hypothesis-generating. The most important modifier in both countries was site. In South Africa, for both primary outcomes, most of the difference seen was due to Site 3. For initiation by 28 days, the absolute RD at Site 3 was 22% (6%–37%). At the other two sites, initiation ≤28 days showed a modest increase of 4%–6%, though with wide CIs. For retention at 8 months, the RD for Site 3 was 15% (1%–29%), while differences at the other sites were small. In Kenya, for initiation ≤28 days, most of the difference seen was due to Sites 1 (11% [2%–21%]) and 3 (7% [0%–15%]), not Site 2, where the difference was small. For our second primary outcome, all three sites differed: Site 1 showed equally poor retention in both study arms; at Site 2, the RD was negative with a wide CI (–13% [–29% to 2%]), while for Site 3, the RD was positive (15% [–2% to 31%]). Other modifiers of effect included sex, age, and reason for clinic visit in South Africa and sex in Kenya, with greater initiation ≤28 days in general for men in both countries. No other variables, including baseline CD4 count, showed an effect modification.

## Discussion

In this randomized evaluation, we found that a simple algorithm for initiating ART in a single visit, without awaiting laboratory tests or additional services, enabled exactly half of HIV-positive adults presenting at primary care clinics in Johannesburg and 70% of this population in Kenya to initiate treatment on the same day. In both countries, roughly half of those had been diagnosed that day. The proportion initiating within 7 days increased by 27% and within 28 days by 10% in South Africa and by 13% within 7 days and 6% within 28 days in Kenya. After 8 months’ follow-up, there was a numerical 6% increase between the arms in retention in care in South Africa and no difference in retention in care in Kenya or known viral suppression in either country.

In much of the world, uptake of ART among those already diagnosed remains far below the global target of 90% [14]. In Gauteng Province, where Johannesburg is located, only an estimated 55% of known HIV-positive persons were on treatment in 2016 [15]. As a result, 42% of AIDS-related deaths nationally were among people diagnosed but not yet on ART that year [16]. In western Kenya, roughly 20% of AIDS-related deaths were estimated to be among those diagnosed but not on ART between 2010 and 2015 [17]. While there are barriers to starting treatment at a number of levels [18], making procedures for ART initiation more efficient—with efficiency encompassing clinical effectiveness, patient behavior, and resource utilization by both providers and patients—is important if high-prevalence countries like South Africa and Kenya are to achieve the 90–90–90 targets for HIV treatment.

Other trials of same-day or accelerated ART initiation have generally reported larger increases in ART initiation, compared to standard care, but similar outcomes after starting ART [4,19–21]. Further details can be found in Ford and colleagues’ 2017 recent review of these studies [19], which informed WHO’s guideline revision in favor of rapid or same-day initiation.

To our knowledge, SLATE is the first algorithm evaluated that does not require technology or infrastructure typically not available in public-sector clinics and is, we believe, simpler to perform than other approaches. For nonpregnant patients, all randomized studies we are aware of to date have relied on POC testing instruments for CD4 staging, TB diagnosis, and/or creatinine clearance, which we have come to believe are not feasible or affordable to place in typical primary health clinics in low- and middle-income countries outside study or demonstration settings. These include, e.g., the RapIT trial, which used POC CD4 counts, TB tests, and creatinine tests [4]; the START trial in Uganda, which relied on POC CD4 counts [20]; and the CASCADE trial in Lesotho, which also utilized an array of POC tests [22]. Unlike the START trial, SLATE attempted no changes to clinic management; unlike CASCADE, it took place entirely in existing facilities. Our hope is that SLATE will provide an alternative that can more readily be implemented in routine care settings, particularly those that currently have the longest delays under standard of care.

In both of the SLATE study countries, most of the benefits of the intervention accrued at a subset of the three study sites. In South Africa, the large improvement in ART initiation at Site 3 was not a surprise to our study team, as Site 3 appeared to be the least efficient of the three sites, with frequent staff turnover and absences, poor procedures for filing records and tracing patients, and long queues and waiting times. Similarly, in Kenya, the intervention did little to improve outcomes at Site 2, which was the best organized of our Kenya sites and initiated 94% of standard-arm patients within 28 days, but it increased ART uptake ≤28 days by 11% at Site 1. It is reasonable to speculate that an intervention like SLATE, which is intended to improve the efficiency of clinic procedures, is most effective at facilities that are least efficient to start with and thus have more room for improvement. If the SLATE intervention were to be rolled out in the study countries, targeting facilities with the worst indicators for placing new patients on treatment, rather than all facilities at once, would thus make sense.

TB symptoms were by far the most common reason for screening out of the SLATE algorithm, though relatively few patients were in fact diagnosed with TB. Persistent headache did not identify any CrAg-positive patients among those with CD4 counts ≤100 who were reflex-tested. Most other reasons for screening out were behavioral rather than clinical, such as prior default from ART or current substance abuse; whether these should trigger referral for additional services before ART initiation is debatable. For many if not most of these patients, the benefits of same-day ART initiation may well outweigh the costs, even for previous defaulters and others who may face adherence challenges.

Although the study was powered to detect an absolute increase of 15% of patients achieving our second primary outcome, from 65% to 80%, the observed increase was a modest 6%, from 48% to 54%, in South Africa, and no improvement was seen in Kenya. Standard care achieved faster ART initiation than expected in both countries. A recent observational study in South Africa estimated that the median interval between diagnosis and initiation fell from 27 to 6 days during this period [23].

The poor postinitiation retention rates in both countries and study arms, even for patients who initiated within 28 days (48% in the standard arm and 54% in the intervention arm for primary outcome 2 in South Africa and 43% in both arms for Kenya) suggest that retaining patients on ART in their first half year of treatment remains a major challenge. Facility support for adherence to and retention in ART varied by site and country and probably also by month. We do not have complete information on what types of postinitiation adherence/retention support were provided nor whether study patients participated in available services because routine data systems did not record uptake of such things as adherence clubs and tracing.

We speculate that for some minority of patients, the offer of same-day initiation simply shifts the point of attrition from before to after starting ART [24]. These patients simply do not wish to be treated, at least at the time of the offer; they may return to care later (and likely sicker) or not at all. The lack of a difference in 8-month retention between the study arms in both countries suggests that the manner of initiation is not in itself the driver of loss to follow-up after initiation. Same-day initiation prompts those who do make it to the clinic at least once to give ART a try, rather than being sent away empty-handed; new interventions will be needed for the critical postinitiation period.

As previously anticipated [7], SLATE had several limitations. First, while the study sites were all typical primary healthcare clinics in South Africa and typical hospital-based HIV clinics in Kenya, they were geographically clustered in each country, making generalizability to the rest of the country uncertain. Second, by necessity, we excluded prior to randomization patients who were not physically or emotionally able to participate, leaving us with a potentially healthier sample than the overall population. Third, because we relied on routine data collection systems for outcomes and follow-up, we had a modest amount of missing data. This was mainly problematic in comparing viral load suppression rates: a majority of patients did not have a viral load test recorded by the 8-month study endpoint because of poor record keeping, nonoperational equipment, or patient or provider decision not to do the routine 6-month test. For the same reason—reliance on routinely collected data for follow-up—we cannot determine what proportion of patients who appear to be lost to follow-up at the 8-month endpoint were in fact undocumented transfers to other healthcare facilities. Fourth, the intervention arm of the study was implemented by trained study staff who achieved near-perfect fidelity to intervention procedures; we might not expect such consistent implementation in routine care settings, and the effect reported may thus not reflect what would be seen in practice. Fifth, participation payments to intervention-arm patients were made after all other study procedures were completed, potentially incentivizing these patients to remain for the full set of procedures. Finally, because this was a pragmatic trial that made no effort to “control” the standard arm, services provided to the standard-of-care comparison arm fluctuated over the enrollment period and by study site. When enrollment into SLATE started, same-day initiation was regarded as a bold and perhaps risky proposition, not addressed in prevailing guidelines; by the time the study ended, roughly a year and a half later, it was a widely accepted practice. As a result, the SLATE algorithm as implemented in this study may be relatively conservative compared to the current (but not former) standard of care.

In conclusion, SLATE demonstrated that South African public-sector, primary healthcare clinics and Kenyan public-sector, hospital-based HIV clinics can feasibly and safely initiate 50%–70% of all new HIV patients onto ART during the patients’ first clinic visit, without the use of expensive POC assays, laboratory results, or additional adherence education or other services for patients. While practice has to some extent caught up with the study—initiation on the same day as diagnosis is now a commonly accepted practice in Kenya [3], South Africa [2], and many other countries—there remains little research on how it should be implemented in a way that maximizes patient benefits. SLATE offers a way to standardize procedures and minimize the burden on eligible patients while still assuring appropriate care for those who need it. Same-day initiation under the SLATE algorithm achieved better uptake of ART in both countries and modest improvement in retention in care at 8 months in South Africa. For both study arms in both countries, though, the proportion of patients achieving the 8-month retention outcome was abysmal. Early retention after initiation, regardless of the speed or manner of initiation, will continue to require additional intervention. For accelerating ART initiation, the next step in is to look more carefully at the large proportion of patients who screened out of the SLATE algorithm to see whether some of those patients too, could be started on ART the same day.

## Supporting information

S1 Checklist CONSORT checklist. (DOCX)

S1 Text Research protocol. (PDF)

S2 Text Further details about data sources and data quality. (DOCX)

S1 Table Details of the SLATE algorithm. SLATE, Simplified Algorithm for Treatment Eligibility.
(DOCX)

S2 Table Effect modification for primary outcomes. (DOCX)

## Abbreviations

ART: antiretroviral therapy
ARV: antiretroviral medication
CI: confidence interval
CrAg: cryptococcal antigen
CRF: case report form
IQR: interquartile range
NASCOP: National AIDS & STI Control Programme
PEPFAR: U.S. President’s Emergency Plan For AIDS Relief
PMTCT: prevention of mother-to-child transmission
POC: point of care
RD: risk difference
RR: relative risk
SLATE: Simplified Algorithm for Treatment Eligibility
TB: tuberculosis
WHO: World Health Organization
