# Supplementary material for: Simplified clinical algorithm for identifying patients eligible for same-day HIV treatment initiation (SLATE): Results from an individually randomized trial in South Africa and Kenya
Source: PLoS Med. 2019 Sep 16;16(9):e1002912. doi: 10.1371/journal.pmed.1002912 (PMC6746347; doi:10.1371/journal.pmed.1002912)
Supplement: S1 Text — (PDF) [file pmed.1002912.s002.pdf]

SLATE

Research Protocol

Randomized Evaluation of a Simplified Clinical Algorithm for Identifying Patients Eligible for Immediate Initiation of Antiretroviral Therapy for HIV (SIMPLIFIED ALGORITHM FOR TREATMENT ELIGIBILITY —THE SLATE STUDY)

BUMC IRB Protocol Number: H-35634 (United States)

Wits University HREC Protocol Number: 160910 (South Africa)

KEMRI SERU Protocol Number: 3408 (Kenya)

WRAIR IRB Protocol Number: 2401 (United States)

Federal-Wide Assurance Numbers:

FWA00000301 (Boston University Medical Campus)
FWA00000715 (University of the Witwatersrand)
FWA 00002066 (Kenya Medical Research Unit)
FWA00000015 (Walter Reed Army Institute of Research)

June 2, 2017

Version 2.1
(Revises previous version 1.1 dated January 13, 2017;
version conforms to INSPIR version number)

Table of Contents

1. Summary **4**
2. Investigators **4**
3. Background **5**
3a. Context: New guidelines for HIV treatment eligibility 5
3b. The problem: loss to follow up before treatment initiation 6
3c. Proposed solution: a standardized clinical algorithm for determining eligibility for immediate treatment initiation 7
4. Objectives **9**
4a. Primary objectives 9
4b. Secondary objectives 9
5. Study Design **10**
5a. Overview 10
5b. Study sites 11
5c. Study population 12
6. Enrollment and Preparation **13**
6a. Pre-screening 13
6b. Screening 13
6c. Consent 14
6d. Pregnancy test 14
6e. Questionnaire 14
6f. Randomization 14
6g. Payment 14
7. Intervention **15**
7a. Algorithm data collection 15
7b. Blood draw 16
7c. Referral of patients with positive screens 16
7d. Dispensing of ARVs and prophylaxis 16
7e. Completion of direct study interaction 17
8. Medical Record Follow Up **17**
9. Data Collection and Management **18**
9a. Sources of data 18
9b. Pre-screening logbook 18
9c. Screening form 18
9d. Case report form 18
9e. Medical record data 19
9f. Study identification numbers and linking of records 21
9g. Data entry and storage 21
10. Staff Supervision and Training **22**
11. Sample Size **22**
12. Outcomes and Data Analysis **22**

12a. Outcomes 22
12b. Analytic methods 23
12c. Dissemination of findings 25
13. Human Subjects Considerations **25**
13a. Risks and protection against risks 25
13b. Benefits 27
13c. Costs and payments to subjects 27
13d. Recruitment 27
13e. Informed consent 28
13f. Protection of confidentiality 28

14. References **28**

12. Appendices **29**

1. Summary

In its 2015 revision of the global guidelines for HIV care and treatment, the World Health Organization called for initiating lifelong antiretroviral treatment (ART) for all patients testing positive for HIV, regardless of CD4 cell count. As countries adopt the new recommendation, known as "treat all," millions of additional patients are becoming eligible for ART worldwide. In sub-Saharan Africa, where most of these patients are located, studies continue to document high losses of treatment-eligible patients from care before they receive their first dose of antiretroviral medications (ARVs). Among facility-level reasons for these losses are treatment initiation protocols that require multiple clinic visits and long waiting times before a patient who tests positive for HIV is dispensed an initial supply of medications. Simpler, more efficient, accelerated algorithms for ART initiation will be needed if "treat all" is to realize the benefits expected.

At a technical consultation convened in October 2015, participants proposed a simplified clinical algorithm to screen patients for eligibility for immediate ART initiation at a patient's first clinic visit, without the use of point-of-care laboratory test technologies. The Simplified Algorithm for Treatment Eligibility (SLATE) uses four screens to assess whether a patient is eligible for immediate (same-day) treatment initiation: i) symptom report, ii) medical history, iii) readiness assessment, and iv) brief physical examination. SLATE is a pragmatic, individually randomized evaluation to determine the effectiveness of the algorithm in increasing ART initiation, compared to standard care, among non-pregnant adult patients. A total of approximately 960 HIV-infected adult patients not yet on ART will be enrolled during a routine clinic visit and randomized to receive the intervention or standard care. Patients in the intervention arm will be administered the SLATE screens; those found eligible under the algorithm will be offered immediate treatment initiation, while those who are not eligible will be referred for standard clinic care. Patients in the standard arm will be referred for ART initiation under standard clinic procedures. All care after the initial visit will be by the clinic under standard of care. The study will be conducted at three healthcare facilities (clinics) in each of two countries, South Africa and Kenya. If successful, SLATE will offer a standardized approach to collecting and interpreting a minimum set of patient data that will avoid delaying treatment initiation for the majority of patients who are eligible for immediate ART, while deferring initiation in the minority who should not start immediately.

2. Investigators

Sydney Rosen
Research Professor
Boston University School of Public Health
Boston, MA, USA
Role on study: principal investigator

Matthew Fox
Associate Professor
Boston University School of Public Health
Boston, MA, USA
Role on study: co-investigator, statistical advisor

Bruce Larson
Research Professor
Boston University School of Public Health

Boston, MA, USA
Role on study: co-investigator, evaluation advisor

Alana Brennan
Research Scientist
Boston University School of Public Health
Boston, MA, USA
Role on study: co-investigator, study manager

Francois Venter
Associate Professor
Department of Medicine
Wits University
Johannesburg, South Africa
Role on study: co-investigator, clinical advisor

Mhairi Maskew
Senior Researcher
Health Economics and Epidemiology Research Office
University of the Witwatersrand
Johannesburg, South Africa
Role on study: South Africa PI, data analyst

Isaac Tsikhutsu
Medical Doctor
Kenya Medical Research Institute/Walter Reed Project HIV Program
Kericho, Kenya
Role on study: Kenya PI

3. Background

3a. Context: New guidelines for HIV treatment eligibility

In its 2015 revision of the global guidelines for HIV care and treatment, the World Health Organization called for initiating lifelong antiretroviral treatment (ART) for all patients testing positive for HIV, regardless of CD4 cell count[1]. This revision followed a series of increases in the global guidelines in the threshold for initiating ART, from a CD4 count of ≤200 cells/mm^3 prior to 2010, to 350 until 2013, to 500 at present. In its recommendation, the WHO cites three anticipated benefits from this approach, which is referred to as "treat all" (alternatively, "test and treat" or "test and start"): reduced morbidity among HIV-infected patients; reduced risk of transmission from HIV-infected individuals to their partners; and "*increases in ART uptake and linkage to care, reduction in the time between HIV diagnosis and ART initiation regardless of baseline CD4 cell count and an increase in the median CD4 value at ART initiation*." A recent analysis concluded that future reductions in HIV-related mortality in sub-Saharan Africa will derive largely from this last benefit, rather than from the first two[2].

Multiple African countries—including South Africa and Kenya, where the proposed study will take place—have already announced their plans to adopt the new guidelines, and it is clear that the trend globally is toward offering ART to all. As this happens, national HIV programs will face the challenge of

initiating individuals newly diagnosed as HIV-infected on ART as efficiently as possible, while ensuring that retention on ART and patient welfare are not jeopardized by the initiation process.

3b. The problem: loss to follow up before treatment initiation

Studies from throughout sub-Saharan African continue to document high losses of treatment-eligible patients from care before they receive their first dose of ARVs. A systematic review of the literature on pre-ART retention in care found that roughly one third of adult patients in sub-Saharan Africa who had already been determined eligible for ART did not start ART within the studies' follow-up periods[3]. If those diagnosed with HIV but not assessed for treatment eligibility had been added, this proportion would have been far higher. Reasons for this include a wide range of facility- and patient-level barriers to initiation[4]. Among facility-level barriers are clinic inaccessibility and inefficiency and initiation protocols designed for the convenience of providers rather than patients. These barriers manifest themselves as multiple required visits, long waiting times, stock outs of supplies, staff absences, and poor communication between staff and patients. Eliminating the eligibility threshold under a "treat all" policy is not likely to solve these problems, as clinics become even more crowded with newly-eligible patients. Simpler, more efficient, accelerated algorithms for ART initiation will be needed if "treat all" is to realize the benefits expected.

In an earlier clinical trial in South Africa (RapIT, H-31880), we demonstrated that same-day treatment initiation, in which all the steps required for ART initiation are performed in sequence during a single clinic visit, significantly increased the proportion of eligible patients started on treatment (RR 1.36, 95% CI 1.24–1.49) and the proportion virally suppressed six months later (RR 1.26, 95% CI 1.05–1.50).[5] The RapIT trial was premised on the notion that standard of care treatment initiation, which in South Africa required 5 or 6 clinic visits over a 1-2 month period, created a burden on patients that deterred many from completing the process. In addition to delaying the start of treatment, each clinic visit entailed transport costs and time, many hours of waiting time in the clinic, and possibly lost wages, child care, and other costs for the patient[6]. The results of the study indicate that accelerating the initiation process does overcome these barriers; in the intervention arm of the study, 75% of study subjects started treatment on the same day as study enrollment, and nearly all the rest within one month. The study demonstrated that it is possible to initiate nearly 100% of eligible patients, and eliminate loss to follow up at this point in the care cascade.

Although RapIT and other studies have shown that the vast majority of patients presenting for HIV care at clinics in Africa can safely start ART on the same day, without delay, there are still patients for whom a delay is necessary. These reasons include:

- Tuberculosis (TB): To minimize the risk of immune reconstitution inflammatory syndrome (IRIS), two weeks of TB treatment is recommended for patients presenting with active TB before ART is started.
- Cryptococcal meningitis (CM): Patients with low CD4 counts should be screened for cryptococcal antigen (CrAg) and given presumptive treatment for CM before ART is started, again to minimize the risk of IRIS.
- Other clinical conditions: HIV patients present with a range of symptoms and conditions that may lead a clinician to decide to delay ART while further investigations or treatment are completed.
- Patient history: Patients who previously defaulted from ART or have a history predicting non-adherence, such as substance abuse, may require additional support before starting ART.
- Patient readiness: Finally, some patients may feel that they are not ready to start treatment on the same day and choose for themselves to delay initiation.

The RapIT trial used multiple rapid, point-of-care laboratory test instruments to generate the laboratory data required for ART initiation in South Africa. The generalizability of its findings is thus limited by the need for expensive equipment that will not be feasible to maintain in most primary healthcare clinics in the region. RapIT also excluded many ART-eligible patients on the basis of clinical trial-specific exclusion criteria that would not apply in a routine care setting. What is needed now is a way to assess eligibility for immediate treatment initiation that is broadly applicable to nearly all patients and does not require either point-of-care laboratory instruments or delays for laboratory results to be received, while screening out patients who should not start treatment immediately for clinical or personal reasons.

3c. Proposed solution: a standardized clinical algorithm for determining eligibility for immediate treatment initiation

In October 2015, the investigators convened a technical consultation of experts in the field to discuss models for accelerating treatment initiation[7]. Participants in that meeting proposed a simplified clinical algorithm to screen patients for eligibility for immediate ART initiation without the use of point-of-care technologies. The simplified algorithm, abbreviated "SLATE," is illustrated in Figure 1.

Figure 1. SLATE algorithm

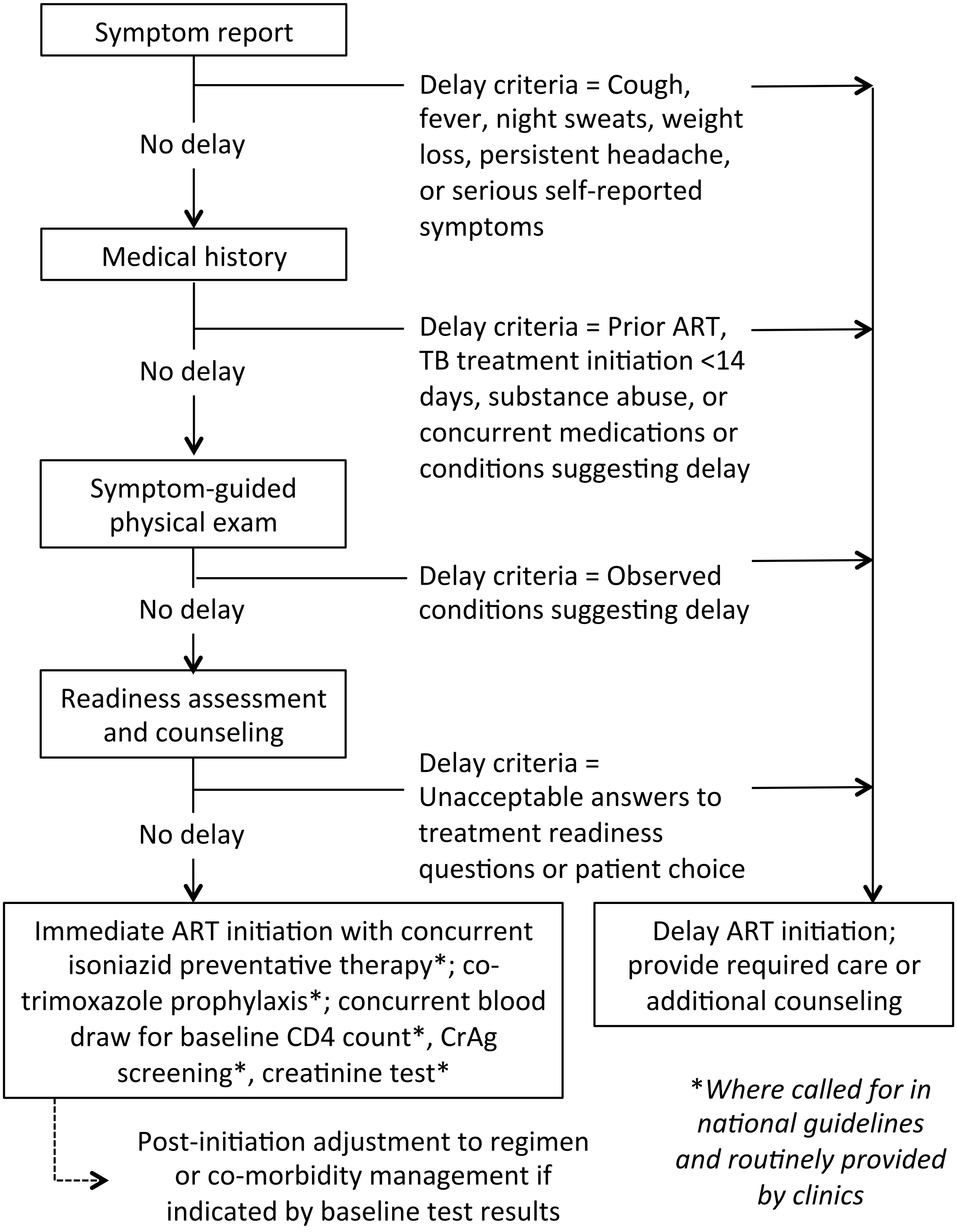

As shown in Figure 1, the SLATE algorithm uses four screens to assess whether a patient is eligible for immediate (same-day) treatment initiation. Each of the screens is intended to identify specific reasons that a patient might *not* be eligible, including current symptoms of opportunistic infections, previous experiences or behaviors that indicate a need for additional treatment readiness or adherence support, and the patient's own concerns about starting treatment. The algorithm captures all components of treatment eligibility screening currently used in routine care in the study countries with the exception of pre-initiation laboratory results. It follows national guidelines for detection of co-morbidities, which vary by country; some countries call for a CrAg test for patients with CD4 counts < 100, for example, while others do not conduct routine CrAg screening.

The purpose of the SLATE algorithm is to create a standardized approach to collecting and interpreting a minimum set of patient data that will avoid delaying treatment initiation for the majority of patients who are eligible for immediate ART, while deferring initiation in the minority who should not start immediately. If successful, it is expected to increase overall uptake of ART and viral suppression in the population of HIV-infected adults eligible for ART in sub-Saharan Africa.

4. Objectives

The SLATE study is a pragmatic, individually randomized evaluation to determine the effectiveness of the SLATE algorithm in increasing ART initiation, compared to standard care, among non-pregnant adult patients. Patients will be individually randomized to either an intervention arm, in which the SLATE algorithm will be implemented, or a standard arm, in which they will receive standard treatment initiation.

4a. Primary objectives

The primary objectives of this study are to:

1. Compare the proportion of study-eligible patients who initiate ART within 28 days of study enrollment between HIV-infected patients offered immediate ART initiation under the SLATE algorithm and patients offered standard ART initiation.

2. Compare the proportion of study-eligible patients who initiate ART and are alive, in care, and retained on ART by eight months after study enrollment between HIV-infected patients offered immediate ART initiation under the SLATE algorithm and patients offered standard ART initiation.

4b. Secondary objectives

The secondary objectives of this study are to:

3. Compare the proportion of study-eligible patients who initiate ART within 14 days of study enrollment between HIV-infected patients offered immediate ART initiation under the SLATE algorithm and patients offered standard ART initiation.
4. At sites where viral load data are available, compare the proportion of study-eligible patients who initiate ART and are virally suppressed eight months after having an HIV test or enrolling in HIV care
5. Compare the proportion of study-eligible patients who initiate ART and are alive, in care, and retained on ART by 14 months and 16 months after study enrollment between HIV-infected patients

offered immediate ART initiation under the SLATE algorithm and patients offered standard ART initiation.

6. Estimate the proportion of HIV-positive patients presenting at study clinics and not yet on ART who are eligible or ineligible for immediate initiation using SLATE algorithm criteria.
7. Describe reasons for ineligibility for immediate initiation, among those found ineligible in the intervention arm.
8. Estimate average time interval in days between study enrollment and ART initiation for each study arm.
9. Describe self-reported patient preferences on the speed and timing of ART initiation.
10. Estimate costs to patients of implementing the SLATE algorithm, compared to standard care.
11. Estimate costs to providers of implementing the SLATE algorithm, compared to standard care.

5. Study Design

5a. Overview

The study will be an individually randomized evaluation that will collect data from consented study subjects using questionnaires, a brief physical examination, a venous blood draw where not already done under routine care, and review of routine medical records. Patients randomized to the intervention arm will be initiated on ART if eligible under SLATE or referred for standard care if not eligible under SLATE. Health facility information that does not pertain to human subjects (e.g. unit costs) will also be collected. Figure 2 summarizes study design and procedures.

Figure 2. SLATE study procedures

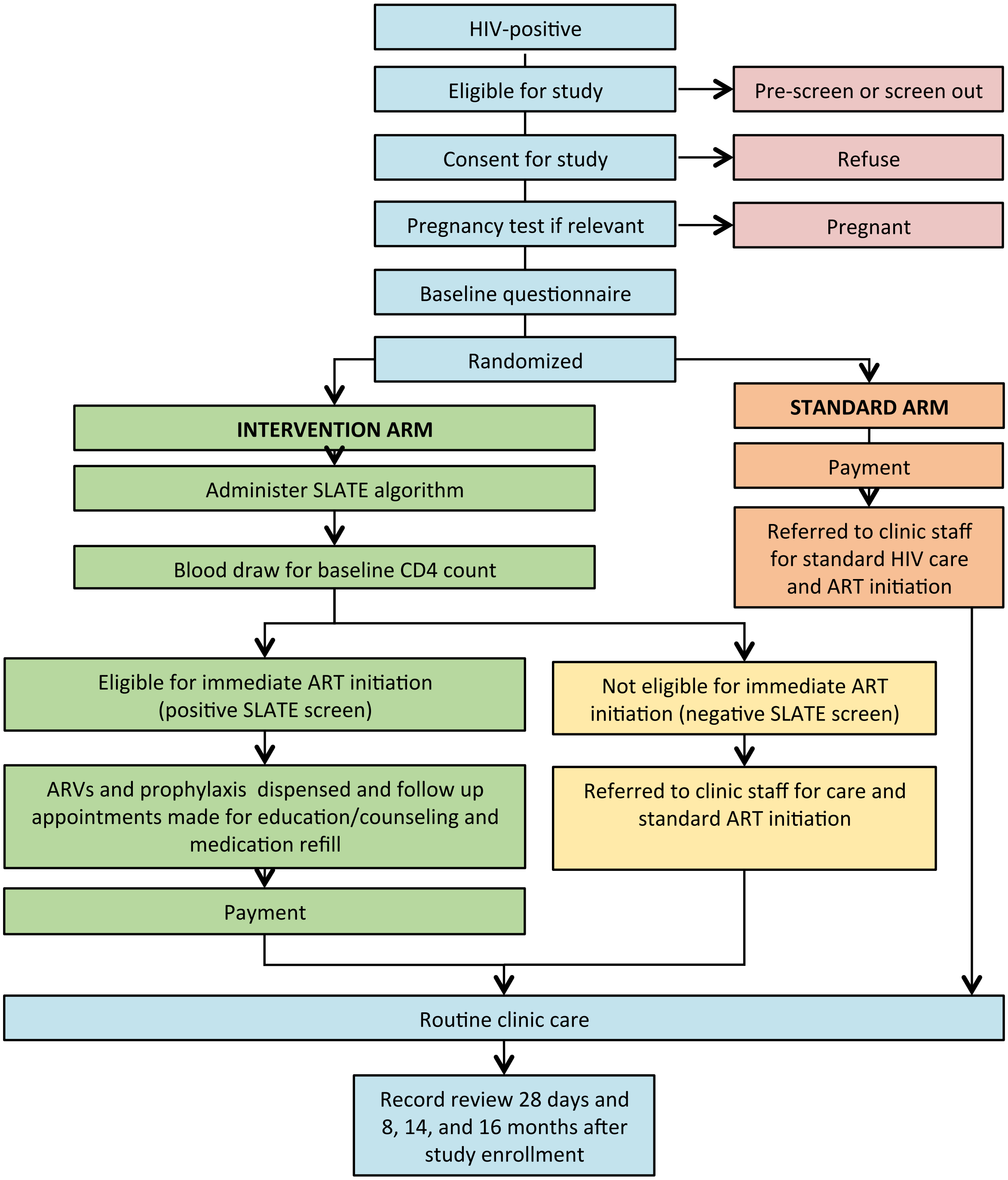

5b. Study sites

The study will be conducted at three high-volume, public sector clinics in each study country. We selected sites that have both a relatively high volume of potentially study-eligible patients, to reduce the time required for enrollment, and have service delivery infrastructure and staff that are typical of the public sector, so that results will be broadly generalizable within each country.

Sites were selected in collaboration with local study teams and in consultation with government health authorities in each area. A letter confirming government authorization for each site to participate in this study will be submitted to the BU and local IRBs prior to study enrollment at that site.

The proposed sites in each country are:

South Africa:

- OR Tambo Primary Health Clinic, City of Johannesburg
- Alexandra Community Health Centre, City of Johannesburg
- Jabulani Dumani Community Health Centre, Ekhurhuleni Metro

Kenya:
- Kericho County Referral Hospital, Kericho
- Kapsabet County Referral Hospital, Kapsabet
- Kombewa County Hospital, Kombewa

5c. Study population

The study population will be all non-pregnant HIV-infected adults who are not yet on antiretroviral therapy.

Inclusion criteria:
- Adult patients (≥18 years) (initiating children on ART is likely to require additional information, making the SLATE algorithm less applicable to pediatric populations)
- Confirmed HIV-positive test result at any time (may have been diagnosed previously)
- Not currently on ART (three-drug combination)
- Presented at the study clinic for any HIV-related reason, including an HIV test, pre-ART monitoring, or ART initiation

Exclusion criteria:
- Pregnant (pregnancy is an exclusion criterion because treatment guidelines for pregnant women differ from those for non-pregnant adults; most pregnant women are diagnosed with HIV and initiated on ART in antenatal clinics, not general adult HIV clinics)
- Not intending to return to this clinic for further HIV care in the coming year (i.e. intends to seek further care somewhere else)
- Not physically, mentally, or emotionally able to participate in the study, in the opinion of the investigators or study staff
- Not willing or able to provide written informed consent to participate in the study
- Previously enrolled in the same study

All the study countries have adopted a policy of “treat all” or “test and treat”, so that all SLATE study subjects will also be eligible for ART under national guidelines. Although implementation of this policy may not be complete and may vary by site, we will enroll all patients who would be eligible under expected treat all guidelines regardless of actual clinic-specific practice at the time of the study. This will

ensure that the SLATE study can generate information for a future in which “treat all” is the standard in most places.

Patients will be followed passively through medical record review for up to 16 months after study enrollment.

6. Enrollment and Preparation

6a. Pre-screening

Patients presenting at a study clinic who are HIV-infected but not yet on ART will be eligible for study pre-screening. This includes patients diagnosed earlier and patients newly diagnosed on the same day. At each study site, the site’s staff (counselors, clerks, and clinical staff, as appropriate to each site) will inform potentially eligible patients about the study at visit registration, which will be defined as the patient’s first point of contact with clinic staff upon arriving at the clinic. Patients who express willingness to learn more about the study will be referred to a study assistant for screening and consent. Patients will be assured that they will not “lose their place” in the clinic queue by agreeing to referral to the study assistant; upon completion of interaction with study staff, the study assistant will escort patients who do not want to participate or who were randomized to standard care to return to their original place in the queue (or front of the line in case their place was lost) to continue with their routine clinic visit.

Where feasible, clinic staff who refer patients to the study assistant will be asked to complete one row in a pre-screening logbook (Appendix 1) for each patient they inform about the study, to allow the study to collect aggregate statistics about the overall patient population and compare it to the screened population. Fields that will be recorded in this logbook are date, sex, age in years, and primary reason for clinic visit (HIV test, CD4 count, pre-ART monitoring, adherence education session, ART initiation). No identifiers of any kind will be collected in this logbook, which is intended to provide only aggregate information. We anticipate that use of the logbook will be feasible at some study sites and some first points of contact at those sites but may not be feasible at others, if clinic staff are too busy or do not remain in a fixed location in the clinic. Where the logbook cannot be used, clinic staff will follow the same procedures for information patients about the study, but no record will be kept. Data analysis requiring pre-screening data will be limited to sites where the logbook is used.

6b. Screening

Upon referral to the study assistant, patients will receive a more complete description of the study, including the details of why it is being done, study procedures, and the need for written informed consent. They will be assured that participation is voluntary and that they can withdraw from the study at any time, without affecting the quality of care provided by the site. They will also be offered the opportunity to ask questions. If the study assistant concludes that all other inclusion/exclusion criteria have been met, patients will then be asked to provide written informed consent to participate, as described below. The study assistant will complete a screening form (Appendix 2) to confirm study eligibility for each patient screened. The screening form will not collect any identifiable information pertaining to individual patients prior to receipt of written informed consent.

Patients will be screened consecutively, as they arrive at the study site. Due to the volume limitations of study staff, however, recruitment may be halted during intervals when the study assistant and/or study

clinician are already busy. We anticipate that clinical data collection will take no more than 30 minutes per patient, allowing us to enroll and collect data from roughly 8-10 patients per day using a single study clinician per site.

6c. Consent

Written informed consent will be obtained from all study participants after screening and prior to study enrollment. The study information sheet and consent form (Appendix 3) will be translated into local languages most commonly spoken by patients at the study sites. Translated consent documents and attestation of consent accuracy will be submitted to the BUMC IRB and local IRB prior to use with any study subjects. Potential subjects will be asked if they would prefer to read the information sheet or have it read to them by the study assistant and will be given the opportunity to ask questions about the study. Those who provide written informed consent will be enrolled. Those who refuse consent will be referred back to the clinic staff to complete their routine visit, and the study assistant will indicate the refusal on the screening form.

6d. Pregnancy test

Because SLATE is not intended for pregnant women, we will conduct a rapid pregnancy test for female patients following consent. Women who have already reported being pregnant will have been screened out prior to consent; the pregnancy test will identify those who were not aware of being pregnant. Patients who have a positive pregnancy test will be withdrawn from the study at this time and escorted to the clinic registration desk to begin antenatal care and PMTCT.

6e. Questionnaire

The study assistant will administer a short questionnaire documenting the subject's basic demographic and socioeconomic characteristics, costs incurred per clinic visit, and preferences regarding ART initiation. As the questionnaire elicits only simple responses and will be completed by the study assistant not the subject, we do not intend to translate the questionnaire itself into subjects' first languages. Instead, for subjects who do not speak English, the assistant will ask the questions in one of the commonly-understood languages used by the study population, and then record the answers in English on the questionnaire. The questionnaire is included in the study case report form in the appendix to this protocol.

6f. Randomization

After the questionnaire has been completed, patients will be randomized to the intervention or standard groups of the study. Randomization envelopes will be generated in blocks of 6 and kept at the study sites. For each enrolled patient, the study assistant will open the next envelope in sequential order, read the randomization group, and record it on the case report form. The study assistant will then inform the patient of the randomization assignment.

Following randomization, standard group patients will be escorted to the clinic registration desk to resume their routine clinic visit. The study assistant will repeat the explanation of next steps in the study, which are limited to review of medical records by the study team, provide study payment as described below, and thank them for their willingness to participate in the study. The study team will have no further personal contact with patients in the standard group.

The study assistant will introduce intervention group patients to the study clinician for algorithm data collection. The study clinician will be a nurse, medical officer, or clinical officer at the same level as the individual responsible for ART initiation under standard care at the study site.

7. Intervention

7a. Algorithm data collection

The SLATE algorithm (illustrated in Figure 1 above) calls for four types of screens: a symptom report, medical history, brief physical exam, and readiness assessment. Each of these is described below and further detail is provided in the case report form appended to this protocol. A negative response to a screen indicates that the patient "screens out" and referall for additional care (consultation, laboratory test, counselling, other) is recommended; if all responses are positive, then the patient "screens" in for immediate initiation under SLATE.

1. Symptom report. Each patient will be screened for current cough, fever, night sweats, and weight loss, which comprise the standard, WHO-recommended TB symptom screen[8], and for persistent (continuous) headache for more two days, which is a symptom of cryptococcal meningitis[9,10], and will be asked about any other symptoms that the patient has noticed.

2. Medical history: To identify individuals who are likely to face problems with medication-taking or adherence, patients will be asked if they have ever been on ART previously, started TB treatment in the past two weeks, are taking concurrent medications for epilepsy or warfarin, which can interact with ARVs, or have a history of substance abuse.

3. Physical exam: A brief, symptom-guided physical exam to look for causes of symptoms or any other evident conditions that could postpone ART initiation will be conducted.

4. Readiness assessment and counselling: A published review of readiness instruments for HIV initiation found that none was notably successful in predicting patient readiness for ART, as indicated by adherence once on treatment[11]. Despite this, there is general consensus that explicit attention to readiness is an important component of treatment initiation. Drawing on the published literature on stage-of-readiness scales[12], we have created a short instrument which we expect to serve primarily as an invitation to patients to raise concerns or questions about treatment readiness with the clinician conducting the assessment. Following the questions, the study clinician will have a brief conversation with the patient to confirm that the patient feels ready to start treatment immediately, understands what happens next, and has no further questions or concerns.

If SLATE were used in routine care, we expect that the screens would be administered sequentially, so that a negative response on the first screen would cause a patient to screen out without having to complete the second screen, and so on. Patients would progress to the next screen only after being found positive on the previous one. For purposes of this study, however, we will administer all four screens, to obtain a complete algorithm data set for each patient in the intervention arm.

We anticipate that for the vast majority of patients, the first three screens will take less than 5 minutes each to administer and the last screen less than 10 minutes. Patients who screen out will require more time, and we therefore estimate needing an average of up to 30 minutes of clinician time per patient in the intervention am.

7b. Blood draw

Cryptococcal meningitis and other co-infections are more likely in patients with very low CD4 counts. Although new national guidelines in the study countries do not require the results of a CD4 count to establish treatment eligibility, most countries will continue to use CD4 counts to monitor patient condition. To avoid asking intervention group patients to wait in the clinic's queue for a blood draw, the study clinician will do a standard venous blood draw, following the same procedures that the clinic would otherwise use. Blood samples will be tested by the laboratories that serve the clinics, following routine procedures, and results will be placed in routine medical records so that the study clinics do not have to repeat the CD4 count for the same patients. The clinic may also use the blood sample for other baseline tests recommended in national guidelines, such as creatinine, hemoglobin, and for patients with CD4 counts under 100 cells/mm^3, cryptococcal antigen (CrAg). The clinic will follow routine procedures for laboratory tests; the study will only be responsible for drawing the required blood sample.

Study staff will monitor CD4 count results for all patients enrolled in the intervention arm. Under national guidelines in both countries, any patient who has a CD4 count under 100 cells/mm^3 and a positive cryptococcal antigen (CrAg) screening result should begin presumptive treatment for cryptococcal meningitis. Results of the CD4 count and CrAg screening test will only be available several days after study enrollment, however. To facilitate proper follow up of any intervention arm patient who does have a positive CrAg screening test, study staff will review CD4 count results for intervention arm patients as soon as the results are available at the study clinics. The study team will alert the clinic to any patient who has a CD4 count under 100 and request that the clinic trace the patient and provide follow up care as guidelines mandate.

7c. Referral of patients who screen out

After completing all four of the SLATE screens, the study clinician will fill out a brief referral letter that reports any substantive findings, such as TB symptoms or a physical exam observation. Intervention arm patients who screen out (have negative responses on any of the SLATE screens) will be referred to routine clinic care to address the issue(s) identified and initiate ART under standard care. The study clinician will discuss and explain the concern(s) with the patient and then escort the patient to the registration desk to continue with a standard clinic visit. The patient will be given the referral letter and encouraged to give the letter to the clinic nurse or medical officer. Patients who do not wish study findings to be shared with clinic staff will not be required to pass on the letter. Patients who are referred to routine care due to a positive SLATE screen will remain in the intervention arm, have the same study follow up as other intervention arm participants, and be analyzed as part of the intervention arm.

For patients who screen in (have positive responses on all the SLATE screens and are eligible for immediate ART initiation) give their agreement, referral letters will be placed in their clinic files along with other source documentation, such as the regular ART record that must be filled out for all new ART patients. Letters will not be filed for patients who do not agree.

7d. Dispensing of ARVs and prophylaxis

Patients who have a full negative SLATE screen (no reasons for delay) will immediately be offered their first supply of ARV medications. While making the offer to dispense, the study clinician will confirm that

the patient still wishes to start ART immediately, answer any questions the patient may have, and reiterate the importance of returning for the patient's next appointment and medication refill.

In addition to ARVs, national guidelines call for prescribing isoniazid preventive therapy (IPT) and/or co-trimoxazole prophylaxis to new HIV patients. The study clinician will also write prescriptions for these medications. Where possible, the study clinician will have a stock of medications in the study room, so that the patient does not need to go anywhere else in the clinic to obtain their medications. If this is not allowed, the study clinician or study assistant will escort the patient to the clinic pharmacy to obtain the medications without waiting in a queue. The clinician or study assistant will then escort the patient to the clinic registration desk to schedule the next appointment, which will include participation in one of the clinic's routine adherence education sessions.

7e. Completion of direct study interaction and payment

Direct study interaction with study subjects will cease as follows:

- *Intervention group (Track 1)*: Upon completion of the ART initiation steps described above, after which subjects will be managed by the study site following standard procedures.

- *Standard group (Track 1)*: after randomization, at which point subjects will be managed by the study site following standard procedures.

From these points on, all subjects, regardless of which type of initiation they received, will be managed by the site following its routine procedures for pre-ART and ART care. Because this study evaluates routine practice and uses retention in care as an outcome, any further study interaction with subjects would affect the results, in particular loss to follow-up. There will therefore be no post-baseline study visits, only routine care visits not involving study personnel. Standard procedures will also be implemented by the study site (not the study team) for study subjects who miss scheduled visits.

The last step in study interaction for both groups will be payment. Reimbursement to consented study subjects of the equivalent of $5-$15 will be provided as a token of appreciate for study participation. The exact amount will be determined in consultation with the study sites and local study teams. The reimbursement will be in the form of cash in Kenya and a voucher to a nearby supermarket and general goods store in South Africa.

8. Medical Record Follow Up

Study subjects in both arms will be followed passively through review of routine medical records kept by the study sites for up to 14 months after study enrollment. This will capture up to 1 month for treatment initiation, up to 12 months of follow up, and up to 1 month for a 12-month routine visit to be made.

The purpose of medical record follow up is to determine which patients enrolled in the study did go on to initiate ART and after what time interval, their early outcomes on treatment, and resource utilization (cost) of initiating ART.

Specific data fields to be collected from medical records are listed under Data Collection below.

9. Data Collection and Management

9a. Sources of data

The study will collect data from five sources, as follows:

1. Pre-screening logbook. Aggregate statistics about the number of potentially eligible patients not screened for the study will be calculated from logbooks completed by clinic staff.

2. Screening form. Screening forms will be completed to confirm study eligibility and consent.

3. Case report form. The study assistant and study clinician will use the CRF to record all information gathered directly from the patient, including the questionnaire and four SLATE screens.

4. Medical records. Routinely collected medical record data will be abstracted from medical records maintained by the study site and by central repositories (e.g. laboratory database) in electronic and paper format.

5. Facility-level records. We will use aggregate information from the study sites to estimate the overall proportion of patients presenting at each clinic who could start ART immediately if SLATE were used in routine care and the resources that would be required.

9b. Pre-screening logbook

As explained above, where feasible we will ask clinic registration staff to complete one row of a pre-screening logbook for each patient who is offered referral to the study assistant for screening. The logbook will have only five fields: date of visit, sex, age in years, primary reason for clinic visit (HIV test, CD4 count, pre-ART monitoring, adherence education session, ART initiation), and whether the referral to the study assistant was accepted (yes/no). No identifiers of any kind will be collected in this logbook, which is intended to provide only aggregate information. Data from the logbook will be used to estimate the total volume of patients who could benefit from SLATE if it were adopted as standard care and to compare the characteristics of patients who do enroll in the study with those of the overall patient population at the site.

9c. Screening form

The study assistant will complete a screening form for each patient referred after pre-screening. The screening form will record whether the patient meets each inclusion and exclusion criterion and provides written informed consent. For patients enrolled, the Study ID number and medical record number will be added to the screening form, which will become part of the case report form for study subjects. For patients who screen out, it will record the reason for ineligibility.

For female patients, the results of the pregnancy test conducted after consent will be recorded on the screening form, as pregnancy is an exclusion criterion for the study. No case report form will be opened for patients who have positive pregnancy tests.

9d. Case report form

The case report form will have eight sections. Sections 1, 7, and 8 will be completed for standard group patients. All sections will be completed for intervention group patients.

1. A short, closed-ended questionnaire to be administered by the study assistant after consent. The questionnaire will elicit basic information about:

    - Patient characteristics (age, sex, marital status, household composition, duration living in current location, education)
    - HIV-related information (familiarity with clinic, time of diagnosis, acquaintance with others with HIV or on ART)
    - Activities and employment
    - Costs to patient of making a clinic visit (transport costs, loss of income, etc.)
    - Patient's preferences regarding ART initiation (single visit, multiple visits, intervals between).

2. Symptom report. The symptom report form will record yes/no to the presence of the symptoms listed above and provide a space for the study clinician to describe or comment on symptoms and to state whether the patient is ineligible for immediate ART initiation due to symptoms.

3. Medical history. The medical history form will record answers to the medical history questions and provide a space for the study clinician to describe or comment on these answers and to state whether the patient is ineligible for immediate ART initiation due to medical history.

4. Physical exam. The physical exam form will record findings of the physical exam and provide a space for the study clinician to describe or comment on these findings and to state whether the patient is ineligible for immediate ART initiation due to the outcome of the medical exam.

5. Readiness assessment. The readiness assessment form will record answers to the readiness questions and provide a space for the study clinician to describe or comment on these answers and to state whether the patient is ineligible for immediate ART initiation due to readiness.

6. Blood draw. The blood draw form will confirm that a blood sample was taken and record the identification number or bar code used to identify the sample on the CD4 count laboratory order form.

7. Patient identifiers. The identification form will capture identifying information about the subject, including name, date of birth, clinic ID number, and national identification number. This information will not be entered into the study database but will be used only for matching CRF and medical record data.

8. Completion form. The last page of the CRF will confirm data collection and close the study visit for the patient.

9e. Medical record data

Once the CRF has been completed, all remaining patient-level data for the study will be collected from routinely maintained medical records. The format of these records will vary by study country and site. Some sites will use electronic medical records, while others will rely on paper-based records and registers or a combination of electronic and paper. We will access all available records for each study

patient, regardless of format or storage location, to try to obtain as complete a study data set as possible. The fields we will aim to capture are listed below.

To be used to link electronic and paper-based records to study identification number only:
- Electronic medical record system number
- Clinic file numbers
- National ID number
- Name
- Date of birth
- Sex

All subjects at clinic visit on date of study enrollment:
- Date of positive HIV test (if available)
- CD4 count (most recent)
- Date and result of TB symptom screen, if done
- Date, type, and result of TB test(s) (current and previous, if any)
- TB treatment, if offered (date of initiation)
- WHO stage and clinical conditions

Subjects who are eligible for ART initiation at the study clinic:
- Reasons for delaying ART initiation, if relevant
- Date ART initiated (first dose of drugs dispensed)
- First-line regimen prescribed

For all clinic visits for duration of study follow up, from study enrollment to the follow-up endpoints indicated above:
- Date of visit
- Primary reason for visit (e.g. pre-ART visit, scheduled medical visit, medication pickup, unscheduled medical visit)
- Types of professionals seen (e.g. nurse, doctor, pharmacy assistant, counselor)
- Group and individual sessions attended (e.g. wellness course, adherence education)
- Numbers and types of all lab tests conducted (including CD4 counts, viral loads, CrAg test, blood tests to determine ARV regimen)
- Other procedures (e.g. x-rays) performed
- Medications prescribed and dispensed (ARV and non-ARV, including isoniazid and cotrimoxazole)
- Admissions for inpatient care since previous visit, if recorded
- Details of inpatient care received (facility, number of days, medications, lab tests, etc.) if recorded

At follow-up endpoints (6 and 12 months after ART initiation or comparable timepoint for those who do not initiate ART):
- Date and result of most recent viral load test
- Status at clinic (in care / died / transferred to another site / stopped care / lost to follow up, defined as > 1 month late for last medication pickup)
- New or recurring HIV-related conditions
- Date and cause of death if applicable
- Date and result of most recent CD4 count

9f. Study identification numbers and linking of records

Upon consenting to participate in the study, each subject will be assigned a random study identification number. This will be a five-digit number in which the first digit indicates the country and the remaining four digits will be a sequential number that does not provide any identifying information.

A password-protected, encrypted linking file will be created in REDCap to link study identification numbers to subjects' names, dates of birth, national identity numbers, and clinic file numbers. These fields are needed to ensure that study ID numbers can be correctly matched to the identification numbers used in the sites medical records. When a subject has been matched to a clinic record, the clinic record number will also be added to the linking file. The linking file will be a separate document from any other study files. Access to the linking file will be limited to the study team, and the file will be stored in a secure location separate from the study database.

9g. Data entry and storage

Screening and CRF data will be entered by study staff onto Android-based tablet computers programmed for data collection using the REDCap mobile app. The REDCap system provides a secure, web-based platform for data entry that allows real time monitoring, querying, and quality control of data by an off-site data manager. It allows access to data to be assigned and restricted to individual study staff as needed and creates audit trails to monitor appropriate access. The mobile app allows data to be entered offline and then uploaded to a central server when an internet connection is available. Records are encrypted upon entry and can be removed from data capturers' tablets once they have been uploaded to the server, reducing the chances of breach of confidentiality if a tablet is lost or stolen. REDCap is supported by the Boston University IT office (https://redcap.bumc.bu.edu/). It has previously been used successfully in South Africa, where the University of the Witwatersrand is a REDCap Consortium partner.

Medical record data for study subjects will be downloaded from electronic medical record systems where available. The lead data analyst will be responsible for ensuring that study identification numbers are correctly matched to the record numbers in the database. In instances where the data analyst cannot match a study ID number to an electronic medical record number using only the fields in the study linking file, a query will be sent to the on-site study staff, who will access the clinic's paper-based patient files to determine whether the subject ever initiated ART and, if so, locate the correct medical record number.

At sites where all or some data are recorded only in individual paper-based files, a note or tab indicating that a patient is a participant in the study will be attached to the cover of each subject's file, to assist site and study staff in locating study subjects' files for data extraction. At regular intervals, files will be pulled and the data fields listed above entered into the study database by study staff. All data entry will take place at the study clinics or other locations where the files are stored; no hard copies of patient records will be taken off site by the study team.

Data will be retained in secure study databases until completion of the study, including completion of all data analysis and report writing associated with the study and closing of the IRB-approved protocol. Study data sets and other electronic files containing study data will then have all identifiers removed

and where possible will be made available for use of other researchers through a repository such as Dryad. Hard copies of study documents will be shredded.

10. Staff Supervision and Training

In each study country, the local study team will include a principal investigator, study coordinator, and site-based study clinicians, assistants, and data capturers. The local PIs will be responsible for training and supervising their teams, under the guidance of the overall study PI and co-investigators. The study clinicians who will implement the intervention will have the qualifications required for ART initiation in each study country. Study assistants who will administer consent and the questionnaire will also be trained as HIV counselors to ensure that they are sensitive to the needs of HIV-positive study subjects.

All members of the study team will be trained in and adhere to the standards laid out in the Belmont Report and NIH guidelines for research and in research ethics applicable to South Africa. Human subjects ethics training will be repeated on an annual basis to ensure that the study team's understanding of research ethics is current.

11. Sample Size

Using results of the RapIT randomized trial we recently conducted in South Africa, we estimate that 65% of treatment-eligible patients will be initiated on antiretroviral therapy and retained on ART in the standard group, and we consider an increase to 80% to be programmatically important. Using an α of 0.05, power of 90%, 1:1 randomization, and an uncorrected Fisher's exact test, this requires a minimum sample size of 197 patients per group, which we will increase to a maximum of 240 per group to ensure sufficient power if patients withdraw or are found ineligible after consent, or 480 per country.

In addition, in South Africa only, we would like to be able to compare between study sites the proportions of patients in the intervention group who screen in and screen out of the SLATE algorithm. Based on early enrollment, we expect the proportion screening in to be a maximum of 67%, which we will conservatively reduce to 60%. We expect the proportion screening in to be a minimum of 32%, which we will conservatively increase to 35%. With an α of 0.05 and power of 90%, we estimate that we will need to enroll a minimum of 79 patients in the intervention arm per site, or a minimum of 158 participants in both arms per site. In order to maintain constant enrollment periods at all three sites in South Africa, we will continue to enroll until the slowest site has reached a minimum of 158. We anticipate that total enrollment at that point will be a maximum of 660 participants in South Africa

The sample size will thus be a maximum of 480 in Kenya and 660 in South Africa, or a maximum of 1140 for the two-country study.

12. Outcomes and Data Analysis

12a. Outcomes

The primary outcomes for the study are:

1. The proportion of patients initiated on ART within 28 days of study enrollment. While national guidelines recommend ART initiation within two weeks of a patient's first clinic visit or HIV diagnosis, recent studies suggest that 28 days is a more realistic deadline for routine care.

2. The proportion of patients who initiate ART and are alive, in care, and retained on ART eight months after study enrollment. Eight months was selected to allow up to 1 month (28 days) to initiate ART, six months of follow up after treatment initiation, and up to 1 month to return for the six-month routine clinic visit.

These outcomes were proposed in a paper published by the investigators based on the MATI technical consultation described in protocol section 3c.[7]

Secondary outcomes will include:

3. The proportion of patients initiated on ART within 14 days of having an HIV test or enrolling in HIV care.
4. The proportion of patients who initiate ART and are virally suppressed eight months of having an HIV test or enrolling in HIV care between HIV-infected patients offered immediate ART initiation under the SLATE algorithm and patients offered standard ART initiation.
5. The proportions of patients who initiate ART and are alive, in care, and retained on ART 14 and 16 months after having an HIV test or enrolling in HIV care. Fourteen months extends the primary outcome endpoint, which is at 8 months, by an additional six months. Sixteen months allows loss to follow up at 12 months to be defined as three months late for the 12-month visit, which is a common endpoint in the literature (16 = 1 month to initiate ART + 12 months' follow up + 3 months to attend 12-month visit).
6. The proportions of HIV-positive patients presenting at study clinics and not yet on ART who are eligible and ineligible for immediate initiation using SLATE algorithm criteria.
7. Reasons for ineligibility for immediate initiation, among those found ineligible in the intervention arm.
8. Average time to ART initiation (days) for each arm.
9. Patient preferences on the speed and timing of ART initiation.
10. Costs to patients of ART initiation under standard and intervention procedures.
11. Costs to providers of ART initiation under standard and intervention procedures.

12b. Analytic methods

We will first conduct descriptive analyses to allow us to determine the characteristics of the sample we have enrolled. We will then analyze the specific outcomes as follows:

Primary outcomes 1 and 2 and secondary outcomes 3, 4, and 5:

For all primary and secondary clinical outcomes, the analytic approach will be the same. All analyses will be by intention-to-treat: subjects will be analyzed according to the intervention they were supposed to receive, whether or not they adhere to the defined intervention. This includes patients randomized to the intervention arm who are screened out of immediate ART initiation by the SLATE algorithm; they will remain in the intervention arm for data analysis. Primary analyses will be pooled across study countries, though we will also conduct stratified analyses by country to look for country-specific differences.

The analysis will begin with a simple comparison of the two treatment groups with respect to baseline predictors of outcomes to look for any imbalances. These potential confounders include demographic

(e.g. age, sex) and socioeconomic (education, distance from clinic, employment, etc.) variables, medications, baseline CD4 counts, and low BMI, among others.

We will then conduct a crude analysis comparing the proportion of patients achieving each dichotomous outcome by group and estimating crude risk ratios and crude risk differences and their corresponding 95% confidence intervals. This will be our primary analysis as we anticipate that randomization of over 1000 subjects should lead to balance in baseline covariates. Should any important imbalances occur by chance, we will proceed with an adjusted model as described below for the country-specific analyses.

Next, we will look for effect modification by important predictors of each outcome. The primary modifier will be country, as practices for care and treatment may differ across the different countries. We will also look for differences in effects stratified by age, sex, baseline BMI, CD4 count, and any other important demographic and clinical predictors of outcomes identified. Our analysis for effect modification will use a simple stratification of the primary analysis by the potential modifier and report crude risk differences and risk ratios and their corresponding 95% confidence intervals.

When stratified by these potential effect modifiers, because the sample sizes within groups will be smaller, it is possible that some baseline covariates may be imbalanced by treatment arm. To adjust for potential differences by baseline covariates, a log-linear regression model will be used to estimate adjusted risk ratios. Variables considered to be important in the univariate stage will be included in multivariate models. Adjusted analyses will include covariates which are unevenly distributed across treatment groups and which could plausibly affect retention or ART initiation. Each of these models can then be used to look for patient-level predictors of treatment uptake and retention in care.

Secondary outcomes 6-9:

Secondary outcomes 6, 7, 8, and 9 are descriptive in nature. These will be described using frequencies, stratified by country, site, and patient characteristics as numbers allow.

Secondary outcome 10:

We will estimate the average cost to patients per clinic visit using questionnaire responses, including transport, substitute labor, and other costs. This cost will then be multiplied by the average number of clinic visits observed in each study arm to generate a total average cost to patients of ART initiation under each model (SLATE and standard). Costs will be reported as means (standard deviations) and medians (IQRs) in local currencies and dollars.

Secondary outcome 11:

At 8 months after study enrollment, all resource usage will be extracted from subjects' medical records and case report forms. Unit costs will be obtained from external suppliers and the site's finance and procurement records and applied to the resource usage data to provide a cost per study patient. Costs will be measured from the provider perspective and will include the cost of all resources utilized for each study patient, including drugs, laboratory tests, outpatient visits, and fixed costs such as building space, equipment, and management staff. We will estimate the average cost to the provider per patient achieving each primary outcome. The average cost per outcome will then be compared between intervention and standard initiation groups to provide an estimate of the cost-effectiveness of the two

strategies. Costs will be reported as means (standard deviations) and medians (IQRs) in local currencies and dollars.

12c. Dissemination of findings

The results of this study will be disseminated as widely as possible in the study countries, where new strategies for increasing the efficiency of treatment initiation are activity sought. A full report will be made to the study sites and other local stakeholders and will be circulated widely and posted on our website. We will also develop a short briefing document to send to the national and provincial departments of health, donor agencies, and other interested organizations, and we will present the results at relevant conferences domestically and internationally. One or more journal manuscripts will be submitted to an appropriate peer-reviewed international journal.

13. Human Subjects Considerations

13a. Risks and protection against risks

For this study, we will not collect any biomedical samples that would not be collected as part of routine care, nor will we implement any clinical procedures that are not routinely carried out under standard of care. The strategy being evaluated merely reorganizes data collection and accelerates the timing of events. The only non-routine data generated for the study itself will be responses to questionnaires. Study staff will conduct a brief physical exam and do a blood draw for intervention arm subjects, but both are procedures that are routinely performed and carry little risk. We therefore believe that our study poses no physical risks to subjects beyond those routinely encountered. It may, however, pose risks associated with loss to HIV care, emotional distress, and breach of confidentiality.

<u>Emotional distress</u>

Risk: By necessity, the study population for this study will include some patients who have been newly diagnosed with HIV. Interacting with them in order to explain the study and confirm eligibility before requesting written informed consent may cause some emotional distress for some potential subjects.

Protection: To minimize any emotional distress caused by interacting with study staff or enrolling in the study, we will train the site's staff to emphasize when they introduce the study to potential subjects that referral to study staff and enrolling in the study are completely voluntary and that those who do not wish to enroll will receive exactly the same care as the study site would otherwise have provided. Potential subjects will also be told that they can discontinue participation at any time, even after consenting. Study staff will be trained to look for signs of emotional distress and instructed to terminate or postpone the enrollment process if subjects appear distraught.

<u>Loss to HIV care</u>

Risk: The study aims to initiate on ART patients who might otherwise be lost to HIV care. Some patients lost before or after ART initiation will seek care at other sites, but many will not and will postpone or end all HIV care and treatment, some until they become more seriously ill, others forever. Although the SLATE strategy is expected to increase the proportion of

patients who do initiate ART, there is a possibility that patients who accept immediate ART initiation under SLATE will subsequently discontinue treatment at a greater rate than do those who receive standard ART initiation. In our prior study of same-day initiation (RapIT), we found that a higher proportion of patients in the intervention arm than in the standard arm were lost to care, but we believe that most of these were patients who otherwise would never have started treatment at all, had only standard initiation been offered. We therefore expect the SLATE intervention to have a net positive impact on our primary outcomes. We cannot be certain of this result in advance, however.

Protection: Because this is an evaluation of routine practice and retention in care is a study outcome, no efforts will be made to retain patients in the study, beyond those already routinely undertaken by the study site clinics as part of standard care. Intervention arm patients will be encouraged to remain in the clinic on the day of study enrollment if a routine ART adherence class or club is available on that day. Study staff will escort intervention arm patients to clinic registration desks to confirm that each patient has made an appointment for a follow-up visit and that the first follow-up visit includes the clinic's regular ART adherence activities (e.g. individual counselling, group education sessions, etc.). Through these efforts, intervention arm patients will have access to the same adherence support as standard arm patients, though the support will be offered after ART initiation rather than before.

<u>Loss of confidentiality</u>

Risk: Because we must collect identifying information in order to link CRF data with medical records, accidental disclosure of HIV status or other loss of confidentiality is also possible. A high level of stigmatization continues to inhibit the disclosure of HIV status in the study populations. Data collected in during the study visit or from clinic records could be disclosed and reveal a person's HIV status.

Protection: Several steps will be taken to protect subjects against the risk of accidental disclosure of HIV status. Individual documents or electronic files (including signed consent forms, case report forms, and the linking file) which could associate patients with an HIV study will be kept strictly confidential. Signed consent forms will be stored in locked cabinets away from the study site, with access limited to the senior investigators. CRFs will be held in electronic versions only and will be password-protected. The linking file will also be password-protected, with access limited to study staff. It will be used only for the purpose of linking study identification numbers with clinic medical records.

To protect against other violations of confidentiality, study staff will be trained in expectations that they are not to disclose any information collected in the study to anyone outside the study team. All study staff will be required to pass an ethics certification course, such as the on-line certification offered by the NIH. All study participants will be encouraged to contact the study clinician or clinic manager to report any undesirable conduct associated with the study. These reports will be brought to the attention of the local and international PI, and appropriate steps will be taken to solve the problems, including reporting to relevant ethical review boards.

13b. Benefits

Direct benefits

Study subjects offered immediate treatment initiation will benefit from the opportunity to start ART quickly and with fewer clinic visits and time delays than they would otherwise have incurred. Study subjects offered standard initiation will not receive any direct benefits from the study but will receive the same care that would have been provided in the absence of the study.

Indirect (societal) benefits

The study has the potential to generate substantial indirect benefits to the subjects. The research we will undertake is expected to reveal whether a simplified clinical algorithm can successfully be incorporated into procedures for ART initiation, leading to more patients initiating ART and a reduction in patient attrition from care. The results of the study may thus lead to improvements in HIV care and keep patients alive and in care longer.

Ratio of benefits to risks

The knowledge to be generated by this study will allow HIV treatment programs to determine whether a simplified clinical algorithm can be used to determine eligibility of immediate ART initiation. This study will address an important gap in the current knowledge around ART initiation and what can be done to reduce the already high proportion of patients who are being lost from care after testing positive. There is currently almost no information available on how to reduce these pre-ART losses. As explained in the previous section, we believe that the risk to subjects in our study is minimal and is outweighed by the indirect benefits and the potential importance of the findings.

13c. Costs and payments to subjects

There will be no costs to subjects for participating in this study.

Subjects who consent to participate and complete study procedures will receive a payment of $10-$15 (local currency equivalent) to reimburse for their time and inconvenience. This payment will be made in the form of a shopping voucher, cell phone air time, or cash, depending on individual study site circumstances.

13d. Recruitment

Identification of potential subjects will occur during patients’ regular clinic visits for any HIV-related service other than monitoring of patients already on ART. At reception and relevant waiting areas at each study site a flier will be posted that informs patients that a study is underway and indicates where to get further information. The flier will be translated into the commonly used languages at the study sites.

The site’s post-test counselors, nurses, and registration staff and the study assistant will inform potentially eligible patients about the study while they are awaiting and/or receiving services. They will explain that a study is underway and that patients found eligible are invited to participate. They will be assured that they will not lose their place “in line” at the clinic and will still receive whatever services

they came to the clinic for. Patients who indicate that they may be interested in participating in the study will be referred to the study assistant for screening.

13e. Informed consent

Written, informed consent will be sought from all study subjects by a trained study assistant. The informed consent information sheet will describe the nature, goals, and procedures of the study and assure subjects their information will be kept confidential. The consent form will explain:

- That patients will not get to decide which group they will be in, but rather that we will decide by chance and they will have a 50-50 chance of being in either group;
- How study procedures will be different from standard care procedures for those randomized to the intervention group;
- How we will do follow-up through medical record data collection; and
- That any individual patient can choose not to participate in the study and be offered standard initiation.

The full informed consent information sheet and form will be translated into the most commonly used languages at the study sites. Translated forms and attestations of translation accuracy will be submitted to the IRB prior to any use of the forms. Subjects who unable to read and/or sign the consent form due to illiteracy will be asked to provide a thumbprint mark, in the presence of a witness who will also sign the form. Patients who do not speak English or any of the languages into which the form has been translated will be regarded as unable to provide written informed consent, which is an exclusion criterion for the study, and will not be eligible for study enrollment.

13f. Protection of confidentiality

Subject data will be captured electronically on case report forms programmed on Android tablet computers using REDCap or another similar, secure application. The tablets will be password-protected and files will be encrypted. When not in use, they will be stored in locked cabinets at the study sites or in study team offices. Study subjects will be assigned sequential study ID numbers upon consent, and data files will contain study ID numbers only, without any other individual identifiers.

A password-protected linking file allowing medical record data to be matched to study-generated data will be maintained in a secure location, with access limited to study staff. Medical record data will be downloaded or extracted from paper records on a regular basis, as needed for data analysis. Electronic medical record datasets may include data on all patients at the study clinic, not just those included in the study, as it may not be possible to select only study participants prior to downloading. As soon as datasets are downloaded, the linking file will be used to select study participants and link their records with their study identification numbers. All identifiers will then be stripped from the clinical data and records for all patients not enrolled in the study will be deleted.

14. References

1. World Health Organization. Guideline on when to start antiretroviral therapy and on pre-exposure prophylaxis for HIV. World Health Organization, Geneva, 2015.
2. Barnabas R, Bendavid E, Bershteyn A, et al. HIV mortality by care cascade stage and implications for

universal ART eligibility. Conference on Retroviruses and Opportunistic Infections, Boston, MA, Feb 22-25, 2016.
3. Rosen S, Fox MP. Retention in HIV care between testing and treatment in sub-Saharan Africa: a systematic review. PLoS Med 2011; 8: e1001056.
4. Lahuerta M, Ue F, Hoffman S, et al. The problem of late ART initiation in Sub-Saharan Africa: a transient aspect of scale-up or a long-term phenomenon? J Health Care Poor Underserved 2013; 24: 359–83.
5. Rosen S, Maskew M, Fox MP, et al. Initiating antiretroviral therapy for HIV at a patient's first clinic visit: the RapIT randomized controlled trial. PLoS Med 2016; 13: e1002015.
6. Lessells RJ, Mutevedzi PC, Cooke GS. Retention in HIV care for individuals not yet eligible for antiretroviral therapy: rural KwaZulu-Natal , South Africa. J Acquir Immune Defic Syndr 2011; 56: 79–86.
7. Rosen S, Fox MP, Larson B, et al. Accelerating the uptake and timing of antiretroviral therapy initiation in sub-Saharan Africa: an operations research agenda. PLoS Med 2016; 13: e1002106.
8. World Health Organization. Guidelines for intensified tuberculosis case-finding and isoniazid preventive therapy for people living with HIV in resource-constrained settings. World Health Organization, Geneva, 2011.
9. Wajanga BM, Kalluvya S, Downs JA, Johnson WD, Fitzgerald DW, Peck RN. Universal screening of Tanzanian HIV-infected adult inpatients with the serum cryptococcal antigen to improve diagnosis and reduce mortality: An operational study. J Int AIDS Soc 2011; 14: 48.
10. Aoussi EF, Ehui E, Demb JP, et al. Cryptoccocal meningitis and HIV in the era of HAART in Cote d'Ivoire. Med Mal Infect 2012; 42: 349–54.
11. Grimes RM, Grimes DE. Readiness: The state of the science (or the lack thereof). Curr HIV/AIDS Rep 2010; 7: 245–52.
12. Rathbun RC, Farmer KC, Lockhart SM, Stephens JR. Validity of a stage of change instrument in assessing medication adherence in indigent patients with HIV infection. Ann Pharmacother. 2007;41: 208–215.

12. Appendices

Appendix 1. Pre-screening log
Appendix 2. Screening form
Appendix 3. Informed consent information sheet and consent form
Appendix 4. Case report form
Appendix 5. Referral letter
Appendix 6. Recruiting flier
