# Supplementary material for: Simplified clinical algorithm for identifying patients eligible for same-day HIV treatment initiation (SLATE): Results from an individually randomized trial in South Africa and Kenya
Source: PLoS Med. 2019 Sep 16;16(9):e1002912. doi: 10.1371/journal.pmed.1002912 (PMC6746347; doi:10.1371/journal.pmed.1002912)
Supplement: S2 Text — (DOCX) [file pmed.1002912.s003.docx]

**S2 Text**

**Further details about data sources and data quality**

Details of data collection procedures are included in the previously published paper describing the SLATE study protocol^[[1]](#footnote-1)^. In this supplementary document we add further information about sources of data and data quality.

The SLATE research team did not conduct any assessment of the validity or reliability of the routine data sets, nor did we directly assist the sites to do so. Data collection and quality control at the study sites were conducted by the clinic staff according to national guidelines and facility level procedures. All the study sites were supported by PEPFAR implementing partners, though the level, duration, and types of support varied and are unknown to us. The implementing partner was the same for all three sites in Kenya but varied in South Africa.

We did triangulate data sources (e.g. paper charts, electronic medical record entries, and laboratory databases) as needed to maximize the completeness of the study data set. For example, if we were not able to trace a patient’s electronic clinic record but the paper chart documented clinic visits or the laboratory database documented viral load test results, these were used to ascertain retention in care. For some data fields, we have more complete data for intervention arm patients because we collected this information during study procedures, rather than relying on the sites’ own procedures. (For example, we have TB symptom prevalence data for all intervention arm patients but few if any standard arm patients.) We also note that because patients were individually randomized within sites, we can be confident that data quality, however high or low, was consistent for both arms of the trial and should not affect comparative results.

In both countries, as shown in Table 2 of the manuscript, a small proportion of patients who did not achieve the second primary outcome were known (reported in site records) to have transferred to other sites, but most were simply reported as lost or defaulted. Data systems did not allow for detection of clinic visits elsewhere in either country. The exception to this is viral load test results, which were collected from laboratory databases that covered the entire country in both Kenya and South Africa. A viral load test thus did not have to originate from the same clinic, as long as it was performed during the specified window (5-8 months after study enrollment). The electronic medical records used in both countries to determine outcomes for the study are limited to HIV-related care; unrelated services (e.g. non-communicable chronic disease or maternal and child health care) delivered at the study site or another facility were not included.

1. Rosen S, Fox MP, Larson BA, et al. Simplified clinical algorithm for identifying patients eligible for immediate initiation of antiretroviral therapy for HIV ( SLATE ): protocol for a randomised evaluation. BMJ Open 2017;7:e016340. [↑](#footnote-ref-1)
