# Supplementary material for: Simplified clinical algorithm for identifying patients eligible for same-day HIV treatment initiation (SLATE): Results from an individually randomized trial in South Africa and Kenya
Source: PLoS Med. 2019 Sep 16;16(9):e1002912. doi: 10.1371/journal.pmed.1002912 (PMC6746347; doi:10.1371/journal.pmed.1002912)
Supplement: S1 Table — SLATE, Simplified Algorithm for Treatment Eligibility. (DOCX) [file pmed.1002912.s004.docx]

**S1 Table. SLATE algorithm details***

| **Screen** | **Overall purpose of screen** | **Reasons for screening out** | **Justification** | **If screen out, anticipated next step** |
| --- | --- | --- | --- | --- |
| Symptom report | Identify self-reported conditions that require additional investigation | Current cough, fever, night sweats, or recent weight loss | These symptoms comprise the WHO-recommended tuberculosis symptom screen[11] | Referral for TB test |
|  |  | Persistent headache for > 2 days | Symptom of cryptococcal meningitis[12,13] | Referral for CrAg screening |
|  |  | Other self-reported symptoms | Other symptoms could indicate the need for further clinical investigation | Referral for additional clinical consultation |
| Medical history | Through self-report identify individuals on concurrent medications or who may struggle with adherence | On ART previously | Patients who have been on ART in the past may require additional adherence counseling | Referral for additional counseling session |
|  |  | Started TB treatment within the past two weeks | Guidelines recommend a two-week delay in ART initiation for patients starting TB treatment | Appointment for ART initiation immediately after the two-week window has passed |
|  |  | Concurrent medications for epilepsy or current warfarin | These medications can interact with ARVs | Referral for additional clinical or pharmacy consultation |
|  |  | Current substance abuse | Use of recreational drugs or over-use of alcohol can create challenges for chronic medication adherence | Referral for additional counseling session |
| Physical examination | Record weight, height, temperature and blood pressure and identify any observable conditions that require additional investigation | Any conditions that call for further investigation prior to ART initiation | Patient may identify previously unreported symptoms or clinician may observe conditions that indicate a need for further clinical investigation before starting ART | Referral for additional clinical consultation |
| Readiness assessment | Confirm that the patient feels ready to start ART today | Responses that indicate reluctance, hesitation, or concerns in starting and adhering to treatment | Creates a structured opportunity for clinician and patient to discuss any concerns that the patient has not yet raised | Referral for additional counseling and follow-up support as indicated |

*Table reproduced from ﻿Rosen S, Fox MP, Larson BA, Brennan AT, Maskew M, Tsikhutsu I, Bii M, Ehrenkranz PD, Venter WDF (2017). Simplified clinical algorithm for identifying patients eligible for immediate initiation of antiretroviral therapy for HIV ( SLATE ): protocol for a randomised evaluation. BMJ Open; 7:e016340. https://doi.org/10.1136/bmjopen-2017-016340
