# Supplementary material for: Simplified clinical algorithm for identifying patients eligible for same-day HIV treatment initiation (SLATE): Results from an individually randomized trial in South Africa and Kenya
Source: PLoS Med. 2019 Sep 16;16(9):e1002912. doi: 10.1371/journal.pmed.1002912 (PMC6746347; doi:10.1371/journal.pmed.1002912)
Supplement: S2 Table — (DOCX) [file pmed.1002912.s005.docx]

**S2 Table. Effect modification for primary outcomes**

| **Stratification variables** | **Outcome: Initiated ART≤ 28 days** | | | | | **Initiated ≤ 28 days and retained at 8 months** | | | | | | | |  |
| --- | --- | --- | --- | --- | --- | --- | --- | --- | --- | --- | --- | --- | --- | --- |
|  | **Standard arm** | **Intervention arm** | **RD**  **(95% CI)** | **RR**  **(95% CI)** | | **Standard arm** | | **Intervention arm** | | **RD**  **(95% CI)** | | **RR**  **(95% CI)** | |  |
|  | **SOUTH AFRICA** | | | | | | | | | | | | |  |
| Original analysis* | 204 (68%) | 232 (78%) | 10% (3 to 17%) | 1.15 (1.04 to 1.27) | 146 (48%) | | 161 (54%) | | 6% (-2 to 14%) | | 1.12 (0.96-1.31) | |  |  |
| *Study site* |  |  |  |  | |  | |  | |  | |  | |  |
| Site 1 (125/124)** | 93 (74%) | 100 (81%) | 7% (-4 to 17%) | 1.08 (0.95 to 1.24) | | 68 (54%) | | 67 (54%) | | 0% (-13 to 12%) | | 1.00 (0.79 to 1.25) | |  |
| Site 2 (90/90) | 75 (83%) | 79 (88%) | 5% (-6 to 15%) | 1.05 (0.93 to 1.19) | | 58 (64%) | | 62 (69%) | | 5% (-9 to 18%) | | 1.07 (0.87 to 1.31) | |  |
| Site 3 (87/84) | 36 (41%) | 53 (63%) | 22% (7 to 36%) | 1.52 (1.13 to 2.06) | | 20 (23%) | | 32 (38%) | | 15% (1 to 29%) | | 1.66 (1.03 to 2.66) | |  |
| *Reason for clinic visit* | |  |  |  | |  | |  | |  | |  | |  |
| HIV test (162/148) | 104 (64%) | 116 (78%) | 14% (4 to 24%) | 1.22 (1.06 to 1.41) | | 73 (45%) | | 76 (51%) | | 6% (-5 to 17%) | | 1.14 (0.90 to 1.44) | |  |
| Any other reason (140/150) | 100 (71%) | 116 (77%) | 6% (-4 to 16%) | 1.08 (0.95 to 1.24) | | 73 (52%) | | 85 (57%) | | 5% (-7 to 16%) | | 1.09 (0.88 to 1.34) | |  |
| *Sex* |  |  |  |  | |  | |  | |  | |  | |  |
| Male (112/109) | 68 (61%) | 82 (75%) | 14% (2 to 27%) | 1.24 (1.03 to 1.49) | | 55 (49%) | | 51 (47%) | | -2% (-15 to 11%) | | 0.95 (0.72 to 1.25) | |  |
| Female (190/189) | 136 (72%) | 150 (79%) | 7% (-1 to 16%) | 1.11 (0.99 to 1.24) | | 91 (48%) | | 110 (58%) | | 10% (0 to 20%) | | 1.22 (1.00 to 1.47) | |  |
| *CD4 count at enrolment* | |  |  |  | |  | |  | |  | |  | |  |
| <200 cells (94/105) | 61 (65%) | 74 (70%) | 5% (-7 to 19%) | 1.09 (0.90 to 1.32) | | 46 (49%) | | 47 (45%) | | -4% (-18 to 10%) | | 0.91 (0.68 to 1.23) | |  |
| 200+ cells (175/183) | 138 (79%) | 153 (84%) | 5% (-3 to 13%) | 1.06 (0.96 to 1.17) | | 96 (55%) | | 111 (61%) | | 6% (-4 to 16%) | | 1.11 (0.93 to 1.32) | |  |
| *Age at enrolment* | |  |  |  | |  | |  | |  | |  | |  |
| <35 years (163/160) | 115 (71%) | 125 (78%) | 7% (-2 to 17%) | 1.11 (0.97 to 1.26) | | 77 (47%) | | 85 (53%) | | 6% (-5 to 17%) | | 1.12 (0.90 to 1.40) | |  |
| 35+ years (139/138) | 89 (64%) | 107 (78%) | 14% (3 to 24%) | 1.21 (1.04 to 1.41) | | 69 (50%) | | 76 (55%) | | 5% (-6 to 17%) | | 1.11 (0.89 to 1.39) | |  |
|  | **KENYA** | | | | | | | | | | | | |  |
| Original analysis* | 210 (89%) | 226 (94%) | 5.6% (1 to 11%) | 1.06 (1.01 to 1.12) | | 136 (57%) | | 137 (57%) | | 0% (-9 to 9%) | | 0.99 (0.85 to 1.16) | |  |
| *Study site* |  |  |  |  | |  | |  | |  | |  | |  |
| Site 1 (90/90)** | 74 (82%) | 84 (93%) | 11% (2 to 21%) | 1.14 (1.02 to 1.27) | | 48 (53%) | | 48 (53%) | | 0% (-15 to 15%) | | 1.00 (0.76 to 1.32) | |  |
| Site 2 (78/81) | 73 (94%) | 74 (91%) | -2% (11 to 6%) | 0.98 (0.89 to 1.07) | | 49 (63%) | | 40 (49%) | | -13% (-29 to 2%) | | 0.79 (0.59 to 1.04) | |  |
| Site 3 (69/69) | 63 (91%) | 68 (99%) | 7% (0 to 15%) | 1.08 (1.00 to 1.17) | | 39 (57%) | | 49 (71%) | | 15% (-2 to 31%) | | 1.26 (0.97 to 1.62) | |  |
| *Reason for clinic visit* | |  |  |  | |  | |  | |  | |  | |  |
| HIV test (109/114) | 98 (90%) | 107 (94%) | 4% (-3 to 11%) | 1.04 (0.96 to 1.13) | | 64 (59%) | | 61 (54%) | | -5% (-18 to 8%) | | 0.91 (0.72 to 1.15) | |  |
| Other (128/126) | 112 (88%) | 119 (94%) | 7% (0 to 14%) | 1.08 (1.00 to 1.17) | | 72 (56%) | | 76 (60%) | | 4% (-8 to 16%) | | 1.07 (0.87 to 1.32) | |  |
| *Sex* |  |  |  |  | |  | |  | |  | |  | |  |
| Male (103/98) | 91 (88%) | 94 (96%) | 8% (0 to 15%) | 1.09 (1.00 to 1.18) | | 61 (59%) | | 55 (56%) | | -3% (17 to 11%) | | 0.95 (0.75 to 1.20) | |  |
| Female (134/142) | 119 (89%) | 132 (93%) | 4% (2 to 11%) | 1.05 (0.97 to 1.13) | | 75 (56%) | | 82 (58%) | | 2% (-10 to 14%) | | 1.03 (0.84 to 1.27) | |  |
| *Baseline CD4* | |  |  |  | |  | |  | |  | |  | |  |
| CD4 < 200 (56/82) | 48 (86%) | 76 (93%) | 7.0% (-4 to 18%) | 1.08 (0.96 to 1.22) | | 32 (57%) | | 47 (57%) | | 0% (-17 to 17%) | | 1.00 (0.75 to 1.35) | |  |
| CD4 > 200 (89/139) | 81 (91%) | 132 (95%) | 4.0% (-3 to 11%) | 1.04 (0.97 to 1.13) | | 50 (56%) | | 77 (55%) | | -1% (-14 to 13%) | | 0.99 (0.78 to 1.25) | |  |
| *Age* | |  |  |  | |  | |  | |  | |  | |  |
| < 35 years (120/111) | 104 (87%) | 105 (95%) | 7.9% (1 to 15%) | 1.09 (1.00 to 1.19) | | 60 (50%) | | 61 (55%) | | 5% (-8 to 18%) | | 1.10 (0.86 to 1.41) | |  |
| 35+ years (117/129) | 106 (91%) | 121 (94%) | 3.2% (-4 to 10%) | 1.04 (0.96 to 1.11) | | 76 (65%) | | 76 (59%) | | -6% (-18 to 6%) | | 0.91 (0.75 to 1.10) | |  |

*Results from Table 2 for the full sample for primary outcomes 1 and 2

**For each stratified analysis, the numbers in (x,x) reports the sample size for the variable for the standard of care arm and the intervention arm.
